# Human induced pluripotent stem cell derived nanovesicles for cardiomyocyte protection and proliferation

**DOI:** 10.1016/j.bioactmat.2025.04.017

**Published:** 2025-05-02

**Authors:** Yuhua Wei, Xiaoxiao Geng, Qing You, Yu Zhang, Fangfang Cao, Gunaseelan Narayanan, Thanh Nguyen, Xiaoyuan Chen, Jianyi Zhang, Lei Ye

**Affiliations:** a Department of Diagnostic Radiology, Yong Loo Lin School of Medicine, National University of Singapore, 1190742, Singapore; b Department of Chemical and Biomolecular Engineering, College of Design and Engineering, National University of Singapore, 1175753, Singapore; c Department of Biomedical Engineering, College of Design and Engineering, National University of Singapore, 1175754, Singapore; d Department of Pharmacy and Pharmaceutical Sciences, Faculty of Science, National University of Singapore, 1175445, Singapore; e Clinical Imaging Research Centre, Centre for Translational Medicine, Yong Loo Lin School of Medicine, National University of Singapore, 1175996, Singapore; f Nanomedicine Translational Research Program, Yong Loo Lin School of Medicine, National University of Singapore, 1175977, Singapore; g Theranostics Center of Excellence (TCE), Yong Loo Lin School of Medicine, National University of Singapore, 1386678, Singapore; h Institute of Molecular and Cell Biology, Agency for Science, Technology, and Research (A∗STAR), 138673, Singapore

**Keywords:** Pluripotent stem cells, Nanovesicles, Cell cycle, Myocardial protection, Regeneration

## Abstract

It remains a significant challenge to reactivate the cell cycle activity of adult mammalian cardiomyocytes (CMs). This study created a hypo-immunogenic human induced pluripotent stem cell (hiPSC) line using clustered regularly interspaced palindromic repeats (CRISPR)/Cas9 gene editing to knockout β2-microglobulin in hiPSCs (^B2MKO^hiPSCs) for manufacturing nanovesicles (^B2MKO^hiPSC-NVs). Approximately 9500 ^B2MKO^hiPSC-NVs were produced from a single ^B2MKO^hiPSC. Proteomic analyses indicated that, compared to ^B2MKO^hiPSCs, the cargos of ^B2MKO^hiPSC-NVs were enriched in spindle and chromosomal proteins, as well as proteins that regulate the cell cycle and scavenge reactive oxygen species (ROS). When administrated to hiPSCs derived CMs (hiPSC-CMs), ^B2MKO^hiPSC-NVs reduced lactate dehydrogenase leakage and apoptosis in hypoxia-cultured hiPSC-CMs through activating the AKT pathway, protected hiPSC-CMs from H_2_O_2_-induced damage by ROS scavengers in the NV cargo, increased hiPSC-CM proliferation via the YAP pathway, and were hypoimmunogenic when co-cultured with human CD8^+^ T cells or delivered to mice. Furthermore, when ^B2MKO^hiPSC-NVs or 0.9 % NaCl were intramyocardially injected into mice after cardiac ischemia/reperfusion injury, cardiac function and infarct size, assessed 4 weeks later, were significantly improved in the ^B2MKO^hiPSC-NV group, with increased mouse CM survival and cell cycle activity. Thus, the proteins in the ^B2MKO^hiPSC-NV cargos convergently activated the AKT pathway, scavenged ROS to protect CMs, and upregulated YAP signaling to induce CM cell cycle activity. Thus, ^B2MKO^hiPSC-NVs hold great potential for cardiac protection and regeneration.

## Graphical abstract

**Figure:**
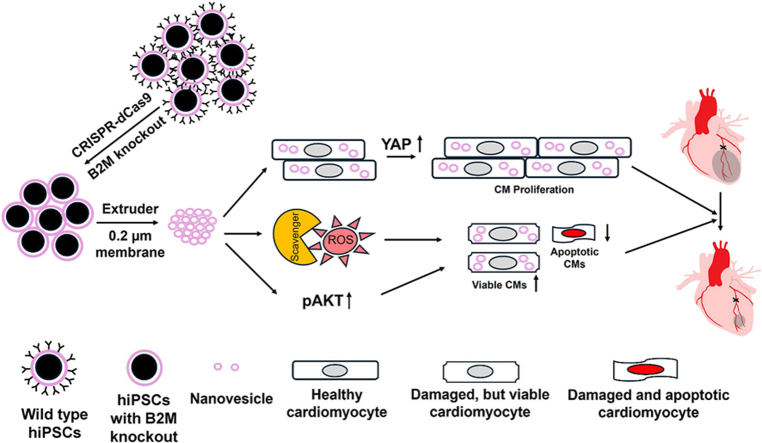

## 1 Introduction

Mammalian cardiomyocytes (CMs) exit the cell cycle shortly after birth; therefore, the CMs of adult mammalian hearts cannot proliferate to repair the damage caused by myocardial infarction (MI) [[1], [2], [3]]. Thus, it remains a significant challenge to reactivate adult mammalian CM cell cycle activity. Human induced pluripotent stem cells (hiPSCs) are robustly proliferative and capable of differentiating into cells of any lineage, but they are also strongly tumorigenic. The cells that exit the site of administration can produce tumors in other organs and tissues [4]. hiPSCs must be fully differentiated into somatic cell lineages before administration, and several efficient protocols for differentiating hiPSCs into CMs have been established [[5], [6], [7]]. Nevertheless, the proportion of transplanted cells that become stably engrafted at the site of administration is exceedingly low, regardless of cell type [[8], [9], [10], [11]], so much of the benefit associated with cell therapy is likely mediated by paracrine factors or extracellular vehicles (EVs) secreted by the engrafted cells. hiPSCs derived EVs (hiPSC-EVs) have been shown to reduce cell death, inflammatory response, and oxidative stress in mouse renal injury models [12], as well as exert anti-fibrotic effects in mouse liver injury models [13]. EVs derived from mouse induced pluripotent stem cells (iPSCs), which are enriched in miRNAs and proteins with proangiogenic and cytoprotective properties, have been demonstrated to reduce myocardial apoptosis, increase left ventricular mass, and improve perfusion in the infarct zone in a mouse model of ischemia/reperfusion (I/R) [14]. In addition, EVs are acellular*,* eliminating the need for cell engraftment, and are unlikely to possess the oncogenic properties associated with direct iPSC administration [14].

However, EVs are generated by living cells and secreted into the culture medium, so yields are low, and the collection and purification procedures are both time- and labor-intensive [15,16]. EVs also present the same antigens expressed by their parental cells [17], and at least one study has indicate that even autologous hiPSC-derived cells may be rejected by the recipient's immune system [18], suggesting that EVs could also stimulate an undesirable immune response after administration.

Unlike EVs, nanovesicles (NVs) are manufactured products generated by extruding whole cells through nano-sized channels. This process allows to produce NVs in clinically relevant quantities, with their contents consisting of the same proteins and nucleic acids that are present in their parental cells [19]. NVs manufactured from MDA-MB-231 tumor cells [19], mesenchymal stem cells (MSCs) [[20], [21], [22], [23], [24], [25]], or hiPSCs [26] have been reported in various studies. These studies typically characterized the profiles of NV cargo [19,20,26] and assessed the function of NVs both *in vitro* [22,24,26] and *in vivo* [21,23,25]. MSCs derived NVs (MSC-NVs) have been shown to promote angiogenesis and neuroprotection *in vitro* [22], attenuate TNF-α induced inflammation in human coronary artery endothelial cells [24], and improve recovery from monocrotaline-induced pulmonary hypertension in rats [23]. MSC-NV administration also promoted CM protection and mitosis in mouse hearts after I/R injury [21], while NVs derived from iron oxide nanoparticles–incorporated MSCs reduced apoptosis and fibrosis, and enhanced angiogenesis and cardiac function recovery in rat hearts after MI [25]. Only one study has shown that hiPSC-NVs promoted angiogenesis and survival of CMs exposed to hypoxia, as well as attenuated cardiac fibroblasts activation *in vitro* [26].

Recognizing that hiPSCs have unlimited capacity for self-renewal and replication, and contain abundant cell cycle molecules and proteins involved in cell function and viability, we aim to use hiPSC-NVs for cardiac protection and regeneration in a mouse model of I/R injury. The anoxia and reperfusion induced oxidative stress damages CMs, often leading to heart failure, as CMs in adult mammalian hearts do not proliferate [27]. Thus, the current study explored the potency and underlying mechanism of hiPSC-NVs for CM protection and cell cycle induction (i.e. cytokinesis) in cultured hiPSCs derived CMs (hiPSC-CMs) *in vitro* and in the hearts of mice after I/R injury *in vivo*, which has not yet been investigated.

Although autologous hiPSC-NVs administration is preferred, ischemic heart disease often occurs alongside comorbid conditions (e.g., diabetes, hypertension, and hypercholesterolemia) that may reduce the efficacy of autologous hiPSC-NVs for myocardial repair and regeneration. To overcome this limitation, we mitigated the immunogenicity of hiPSC-NVs by knocking out the expression of β2-microglobulin (B2M) in hiPSCs (^B2MKO^hiPSCs) before manufacturing the NVs. This modification enabled ^B2MKO^hiPSCs derived NVs (^B2MKO^hiPSC-NVs) to evade clearance by cytotoxic CD8 T lymphocytes after administration [28].

## 2 Materials and methods

### 2.1 Generation and characterization of ^B2MKO^hiPSCs

^B2MKO^hiPSCs were generated from an established hiPSC line [7,8,[29], [30], [31]] via CRISPR/Cas9 gene editing. The original hiPSC line was reprogrammed from commercial neonatal human dermal fibroblasts (ATCC, USA) using the non-integrating Sendai virus carrying four reprogramming factors: OCT4, SOX2, KLF4, and C-MYC [7]. hiPSCs were cultured in Matrigel-coated culture dishes with mTeSR Plus medium (Stemcell Technologies, Canada) until 70 % confluence, then dissociated with TrypLE solution (Gibco, USA). For CRISPR editing, cells were incubated with 37.5 pM total sgRNA (Integrated DNA Technologies, Singapore), 1 μg *sp*Cas9 protein (TrueCut™ Cas9 Protein v2; Invitrogen, USA), and 1.25 μL R buffer (Neon transfection kit, Invitrogen, USA) for 20 min at room temperature to form ribonucleoprotein complexes. These complexes were electroporated into cells using the Neon transfection system (Invitrogen, USA) as directed by the manufacturer's instructions. The 198- or 209-bp sequences were excised from exon 2 of the B2M gene using sgRNA spacer sequences (ACAAAGTCACATGGTTCACA and TCACGTCATCCAGCAGAGAA). After electroporation, cells (500–5000 cells/cm^2^) were seeded onto Matrigel-coated 6-well plates and cultured in mTeSR Plus for 5 days. Individual colonies were then transferred into separate wells of a 24-well plate and expanded for one week before genomic sequencing. Genomic DNA was isolated using Quickextract™ DNA extraction solution (QE09050, Lucigen, UK), and PCR and Sanger sequencing of the region of interest were performed with appropriate primers (Supplemental Tables 1 and 2) to identify clones of ^*B2MKO*^*hiPSCs*. Potential off-target sites, as predicted by Benchling, were also sequenced to confirm the absence of any off-target edits. Sequencing data were analyzed using ICE Analysis v2.0 (https://ice.synthego.com/#/). Both ^B2MKO^hiPSCs and wild-type hiPSCs (^WT^hiPSCs) were maintained in a feeder-free system with mTeSR and passaged every 4–5 days.

Karyotyping was assessed as described previously [30]. The pluripotency of ^B2MKO^hiPSCs was characterized via fluorescence immunostaining and teratoma formation assays. For fluorescence immunostaining, hiPSCs were fixed with 4 % paraformaldehyde for 20 min at room temperature, permeabilized with 0.1 % Triton X-100, and blocked with Ultra-V block (Thermo Fisher, USA). The cells were then incubated overnight with primary antibodies (1: 100 dilution) of mouse anti-Oct3/4 or anti-SSEA-4 (SC-365509 or SC-21704; Santa Cruz, USA) in PBS containing 10 % goat serum (10 % PBS) overnight at 4 °C. On the second day, samples were incubated with PE-conjugated donkey anti-mouse IgG antibody (Jackson ImmunoResearch, USA) in 10 % PBS for 1 h at room temperature. The cells were labeled with 4′,6-diamidino-2-phenylindole (DAPI), washed, and viewed under an Olympus fluorescence microscope. Teratoma formation was assessed by injecting 2 × 10^6 B2MKO^hiPSCs into the flanks of NOD-SCID mice. The teratomas were explanted two months later [30], embedded in paraffin, sectioned, stained with hematoxylin and eosin, and examined under an Olympus microscope to identify endodermal, mesodermal, and ectodermal cells.

Human leukocyte antigen class-I (i.e., HLA-I-A/B/C, HLA-I-E, and HLA-I-G), -II (i.e., HLA-II-DR/DQ/DP), and CD47 (Supplemental Table 2) expressions on hiPSCs were evaluated by flow cytometry [29,32].

### 2.2 Generation and characterization of ^B2MKO^hiPSC-NVs

^B2MKO^hiPSCs were cultured to approximately 80 % confluence, harvested with Versene, washed with DPBS, frozen in CryoStor (Stem Cell technologies, Canada), and stored at −80 °C. For ^B2MKO^hiPSC-NV production, ^B2MKO^hiPSCs were washed three times with DPBS, resuspended in 5 mL DPBS, and passed through a mini-extruder (610000, Avanti Polar Lipids, USA) with a 0.2-μm filter membrane. The NV-containing supernatants were centrifuged at 1200 RPM at 4 °C for 5 min to remove cellular debris. The supernatants were then diluted with DPBS to a final volume of 35 mL and ultracentrifuged at 24,000 RPM (55,768 g, 50 V39 rotor) using the Sorvall WX + Ultra Centrifuge (Thermo Scientific, USA) and 4 °C for 2 h. NV pellets were resuspended in 1 mL DPBS or 0.9 % NaCl, passed through a 0.22-μm syringe filter, and stored at −80 °C. NV size and concentration were measured using the Nanosight NTA3.0 sub-micro particle imaging system (Malvern, USA), and NV morphology was imaged using a Technia T12 Spirit electron microscope. The zeta potential of the ^B2MKO^hiPSC-NVs was measured using the ZETASIZER Nano series (Malvern, England).

To compare the efficiency of hiPSC-NV production yield with that of hiPSCs secreted EVs (hiPSC-EVs), ^B2MKO^hiPSCs at 80 % confluence were washed with DPBS and cultured in TeSR-E8 for 24 h. The cell number was counted, and the supernatant was collected and ultracentrifuged at 24,000 RPM at 4 °C for 2 h. EV pellets were resuspended in 1 mL DPBS, passed through a 0.22-μm syringe filter, and stored at −80 °C. EV size and concentration were measured using a Nanosight.

To determine the tumorigenic risk of hiPSC-NVs, 3 × 10^11 B2MKO^hiPSC-NVs and 2 × 10^6 B2MKO^hiPSCs were injected into the left and right flanks of NOD-SCID mice, respectively. The mice were observed for up to 2 months.

### 2.3 Uptaken of ^B2MKO^hiPSC-NVs by hiPSC-CMs

To visualize the uptake of ^B2MKO^hiPSC-NVs, NVs in the supernatant were mixed with 1 mL of dilute C containing 4 μL of the PKH67 (MINI67, Sigma-Aldrich, USA) dye for 5 min, followed by the addition of 2 mL of fetal bovine serum to stop the reaction. Then NVs were harvested as described above. hiPSC-CMs were cultured with PKH67-labeled ^B2MKO^hiPSC-NVs for 24 h, and images were captured using an Olympus IX83 fluorescence microscope.

To determine whether ^B2MKO^hiPSC-NVs would be directly internalized into lysosomes by the cells, hiPSC-CMs were incubated with PKH67 labeled ^B2MKO^hiPSC-NVs for 24 h, followed by incubation with LysoView™ 550 (Biotium, USA) as directed by the manufacturer's instructions. Images of LysoView and PKH67-labeled ^B2MKO^hiPSC-NVs in hiPSC-CMs were captured using an Olympus confocal fluorescence microscope and analyzed using JACoP Plugin of ImageJ software. The results from the Manders' Coefficients (using thresholds) were used to calculate the percentages of ^B2MKO^hiPSC-NVs overlapping lysosomes or lysosomes overlapping with ^B2MKO^hiPSC-NVs.

### 2.4 hiPSC-NV immunogenicity

To determine whether B2M knockout could reduce the immunogenicity of NVs, ^WT^hiPSC derived NVs (^WT^hiPSC-NVs) and ^B2MKO^hiPSC-NVs were either co-cultured with human CD8^+^ T cells *in vitro* or injected into C56BL mice *in vivo*. Human CD8^+^ T lymphocytes (200-0164, Stem Cell Technologies) were cultured in blood cell growth medium (615-250, Sigma-Aldrich), either alone or supplemented with 5 × 10^9 WT^hiPSC-NVs or ^B2MKO^hiPSC-NVs for 24 h at 37°C. Afterward, CD8^+^ T cells were harvested and stained with anti-CD38 and anti-CD69 antibodies for 30 min at 4°C (Supplemental Table 3). The proportion of CD8^+^ T cells expressing either CD38, CD69, or co-expressing both CD38/CD69 was determined using a BD LSR Fortessa flow cytometer.

*In vivo*, C57BL mice were anesthetized with 2–2.5 % isoflurane, and the site of intraperitoneal abdominal injection was disinfected. A total of 0.2 mL of 0.9 % NaCl, with or without 3.2 × 10^11 B2MKO^hiPSC-NVs or ^WT^hiPSC-NVs*,* was injected. One and three days later, mice were anesthetized with isoflurane, and 100 μL of blood was drawn from the retro-orbital plexus. The blood was mixed with 9 × volume ACK lysing buffer (A10492-01, Gibco, USA) to remove red blood cells, and the remaining mononuclear cells were washed three times with 2 % PBS, then resuspended in 2 % PBS. The cells were stained with anti-CD3, anti-CD4, anti-CD8, and anti-CD69 primary antibodies (Supplemental Table 3) for 30 min at 4 °C. After washing with 2 % PBS, the cells were resuspended in 2 % PBS and analyzed using a BD LSR Fortessa flow cytometer [32,33].

### 2.5 Proteomic analysis of ^B2MKO^hiPSCs and ^B2MKO^hiPSC-NVs

^B2MKO^hiPSCs were cultured until 80 % confluence, harvested, and washed with DPBS. The cells were then either lysed in 6 M Urea buffer or used to produce ^B2MKO^hiPSC-NVs, which were also lysed in 6 M Urea buffer. Protein concentrations were determined using the Bradford Quantification method for lysates from ^B2MKO^hiPSCs, and the proteomic profiles were analyzed by Novogene Corporation Inc., (USA).

***Trypsin treatment*:** Each protein sample was digested with trypsin, mixed with formic acid, and the pH was adjusted to below 3. The samples were then centrifuged at 12,000 g for 5 min. The supernatant was loaded onto a C18 desalting column, washed with washing buffer (0.1 % formic acid, 3 % acetonitrile), and eluted with elution buffer (0.1 % formic acid, 70 % acetonitrile). The eluates from each sample were collected and lyophilized.

***Data-dependent acquisition spectrum library construction:*** Data-dependent acquisition **(**DDA) spectrum library construction and data-independent acquisition (DIA) mode identification using UHPLC-MS/MS were performed by Novogene Corporation Inc., (USA).

***Separation of fractions:*** Mobile phases A (2 % acetonitrile, pH adjusted to 10.0 using ammonium hydroxide) and B (98 % acetonitrile, pH adjusted to 10.0 using ammonium hydroxide) were used to develop a gradient elution. The lyophilized powder was dissolved in mobile phase A and centrifuged at 12,000 g for 10 min at room temperature. Each sample was fractionated using a C18 column (Waters BEH C18, 4.6 × 250 mm, 5 μm) on a Rigol L3000 HPLC system, with the column oven set to 45 °C. The eluate was monitored at UV 214 nm, collected at one tube per minute, and then combined into 4 or 6 fractions. All fractions were dried under vacuum and reconstituted in 0.1 % (v/v) formic acid (FA) in water.

#### 2.5.1 LC-MS/MS analysis in DDA mode

***EASY-nLC*^*TM*^*-HFX:*** Mobile phases A (0.1 % FA in H_2_O) and B (0.1 % FA in 80 % ACN) were used to develop a gradient elution. For transition library construction, shotgun proteomics analyses were performed using an EASY-nLC™ 1200 UHPLC system (Thermo Fisher, Germany) coupled with a Q Exactive™ HF-X mass spectrometer (Thermo Fisher, Germany) operating in DDA mode. A half sample, containing 4 μg of fraction supernatant and 0.8 μL of reagent, was injected into a homemade C18 Nano-Trap column (4.5 cm × 75 μm, 3 μm). Peptides were separated using a homemade analytical column (15 cm × 150 μm, 1.9 μm), and a linear gradient elution method. The separated peptides were analyzed using the Q Exactive™ HF-X mass spectrometer (Thermo Fisher), with a Nanospray Flex™ ion source. The spray voltage was set to 2.1 kV, and the ion transport capillary temperature was set to 320 °C. Full scans were performed with a range of *m*/*z* 350 to 1500, a resolution of 120,000 (at *m*/*z* 200), an automatic gain control (AGC) target value of 3 × 10^6^, and a maximum ion injection time of 80 m s. The top 40 precursors with the highest abundance in the full scan were selected and fragmented by higher energy collisional dissociation and analyzed in MS/MS mode with a resolution of 15,000 (at *m*/*z* 200), an AGC target value of 5 × 10^4^, a maximum ion injection time of 45 m s, a normalized collision energy of 27 %, an intensity threshold of 1.1 × 10^4^, and a dynamic exclusion of 20 s. The raw MS data o files were saved as “.raw” and used to construct the DDA spectrum library.

***nanoElute-timsTOF Pro2:*** Mobile phase A (100 % water, 0.1 % formic acid) and B (80 % acetonitrile, 0.1 % formic acid) were prepared. The lyophilized powder was dissolved in 10 μL mobile phase A and centrifuged at 14,000 g for 20 min. Shotgun proteomics analysis was performed using a nanoElute UHPLC system (Bruker, Germany) coupled with a timsTOF Pro2 mass spectrometer (Bruker, Germany) operating in DDA mode. Peptides were separated using a homemade analytical column (25 cm × 75 μm, 1.6 μm) with linear gradient elution. The separated peptides were analyzed by the timsTOF Pro2, with a Captive Spray ion source and a spray voltage of 1.5 kV. Full scans were performed with a range of *m*/*z* 100 to 1700. The ramp time was 100 m s, and Lock Duty Cycle was set to 100 %. PASEF settings were as follows: 10 MS/MS scans during 1.17 s, ion intensity cutoff was set to 2,500, and the scheduling target was set to 10,000. The raw MS data files were saved as “.d”.

#### 2.5.2 LC-MS/MS analysis in DIA mode

***EASY-nLC*^*TM*^*-HFX:*** Mobile phases A (0.1 % FA in H_2_O) and B (0.1 % FA in 80 % ACN) were used to develop a gradient eluant. A half sample, containing 4 μg fraction supernatant and 0.8 μL reagent, was injected into the EASY-nLC™ 1200 UHPLC system, which was coupled with an Q Exactive™ HF-X mass spectrometer operating in DIA mode. The system used a spray voltage of 2.1 kV, Nanospray Flex™ ion source, and a capillary temperature of 320 °C. For DIA acquisition, the *m*/*z* range was set from 350 to 1500. The MS1 resolution was set to 60,000 (at *m*/*z* 200), with an AGC target value of 5 × 10^5^ and a maximum ion injection time of 20 m s. Peptides were fragmented by higher energy collisional dissociation in MS2, in which the resolution was set to 30,000 (at 200 *m*/*z*), with an AGC target value of 1 × 10^6^, and a normalized collision energy of 27 %. The raw data from the MS detection was saved as “.raw”.

***nanoElute-timsTOF Pro2:*** Mobile phase A (100 % water, 0.1 % formic acid) and mobile phase B (80 % acetonitrile, 0.1 % formic acid) were prepared. The lyophilized powder was dissolved in 10 μL mobile phase A and centrifuged at 14,000 g for 20 min. Shotgun proteomics analysis was performed using the nanoElute UHPLC system, which was coupled with a timsTOF Pro2 mass spectrometer operating in the DIA mode. Peptides were separated using a homemade analytical column (25 cm × 75 μm, 1.6 μm) with linear gradient elution. The separated peptides were analyzed by the timsTOF Pro2, with a Captive Spray ion source and a spray voltage of 1.5 kV. The full scan range was set from *m*/*z* 100 to 1700. The ramp time was 100 m s, and the Lock Duty Cycle was set to 100 %. The window size was 25 Da, and the number of mobility windows was 2. The raw data from the MS detection was saved as “.d”.

#### 2.5.3 Proteomic data analysis

Raw DDA data were analyzed using Spectronaut-Pulsar (Biognosys AG, Switzerland) with default settings for initial peptide identification. A total of 87,682 peptides and 9385 proteins were identified after searching the homo_sapiens_uniprot_2021_7_15.fasta.fasta (202160 sequences) database with trypsin designated as the digestion enzyme. The mass tolerance of the precursor ion was 10 ppm and mass tolerance of the product ion was 0.02 Da. Carbamidomethylated cysteine residues were specified as the fixed modification, acetylation was specified as the N-Terminal modification, and oxidation of methionine was specified as a dynamic modification. A maximum of 2 missed cleavage sites was allowed, and peptide-spectrum matches (PSMs) with a reliability >99 % were designated as valid. Each identified protein contained at least one unique peptide. PSMs and protein levels with false discovery rates (FDR) not exceeding 1.0 % were retained. Mixed peptides were processed in DDA mode to generate the proteomic library and produce the ion-pair chromatographic peaks.

Raw DIA data were analyzed using Spectronaut-Pulsar (Biognosys AG, Switzerland), employing ion-matching and peak-area calculations for both qualitative and quantitative identification. A total of 81,108 peptides and 8304 proteins were identified. Item response theory was applied to the sample to correct for retention time, and the precursor ion Q-value cutoff was set to 0.01. Identified PSMs and protein levels with false discovery rates not exceeding 1.0 % were retained. Decoy generation was performed using a mutation-based strategy, similar to scrambling, which randomly swaps amino acid positions (minimum = 2, maximum = half of the peptide length). The standardization strategy was set to global normalization. Statistical analysis of protein quantification was performed using the T-test.

Heatmap analysis was performed using the pheatmap package (https://CRAN.R-project.org/package = pheatmap). Functional annotation was carried out using blast2GO version 5, and gene ontology (GO) enrichment analysis was performed with GOATOOLS. The Kyoto Encyclopedia of Genes and Genomes (KEGG) signaling pathway analysis was conducted using the KEGG mapper (https://www.kegg.jp). The Voronoi overview of signaling pathways was analyzed using Reactome (https://reactome.org). Protein-protein interaction (PPI) networks were generated using STRING (https://cn.string-db.org) and visualized in Cytoscape (https://cytoscape.org/). The proteomics data have been deposited to the ProteomeXchange Consortium via the PRIDE [34] partner: PXD062056.

#### 2.5.4 *Western Blot analysis to validate proteins which are more abundant in*^*B2MKO*^*hiPSC-NVs than in*^*B2MKO*^*hiPSCs*

^B2MKO^hiPSCs and ^B2MKO^hiPSC-NVs were lysed using M-PER Mammalian Protein Extraction Reagent. Total protein concentrations were determined using the Bradford Quantification method. Proteins were separated on a 4–20 % Mini-PROTEAN® TGX Precast Protein Gels (Bio-Rad, USA) and subsequently transferred to a polyvinylidene difluoride (PVDF) membrane. After blocking with 5 % non-fat milk in Tris-buffered saline with Tween-20 (TBST), the blots were incubated with primary antibodies (Supplemental Table 4) overnight at 4 °C: PRDX2, PRDX6, GSR, TPR, SKA2, CBX5, INCENP, RBBP4/7, CKS1/2, Ki67, and RIF1. Detection was performed using goat anti-rabbit or anti-mouse IgG conjugated with horseradish peroxidase (HRP). The binding of antibodies was visualized using the SuperSignal West Femto Maximum Sensitivity Substrate (34095, Thermo Scientific) and captured with the ChemiDoc™ MP System (Bio-Rad, USA). Protein expression levels were normalized to GAPDH and expressed as percentages of GAPDH.

### 2.6 Cytoprotection of ^B2MKO^hiPSC-NVs on hiPSC-CMs under hypoxia

***The lactate dehydrogenase (LDH) assay***: the cytoprotective effect of freshly prepared and cryo-preserved (stored at −80°C for 3 weeks) ^B2MKO^hiPSC-NVs was assessed. hiPSC-CMs were cultured in 12-well plates and washed three times with DPBS. Cells were, then, cultured in 500 μl Hanks balanced salt solution (HBSS), supplemented with or without 6.25 × 10^7^ to 10 × 10^9 B2MKO^hiPSC-NVs/mL, and incubated under hypoxic conditions (4.5 % O_2_, 5 % CO_2_, and 90.5 % N_2_) for 24 h ^8, 9^. The supernatant was collected to determine LDH intensity using the Cytotoxicity Detection Kit (Roche, USA), following the manufacturer's instructions.

***The cell counting Kit-8 (CCK-8) assay:*** to assess cell viability after hypoxia and treatment with ^B2MKO^hiPSC-NV, hiPSC-CMs were cultured in 48-well plates with 200 μL HBSS, supplemented with or without 6.25 × 10^7^ to 10 × 10^9 B2MKO^hiPSC-NVs/mL, and incubated under hypoxic conditions (4.5 % O_2_, 5 % CO_2_, and 90.5 % N_2_) for 24 h ^8, 9^. After incubation, 20 μL CCK-8 buffer (AR1160, Boster Bio) was added to each well, and cells were incubated at 37°C for 4 h. The absorbance of the supernatant was measured at 450 nm using a TECAN microplate reader. Results were presented as percentages, after normalized to the measurement in the supernatant without ^B2MKO^hiPSC-NV treatment, which was considered 100 %.

***Terminal deoxynucleotidyl transferase dUTP nick end labeling (TUNEL) assessment of apoptosis***: to visualize apoptotic cells, hiPSC-CMs subjected to hypoxia were assessed using the In situ Cell Death Detection Kit (Roche, Germany) ^8, 9^. Briefly, cells were fixed, permeabilized, and incubated with 10 % PBS containing a 1:100 dilution of rabbit anti-cardiac troponin T (cTnT, ab91605, Abcam, USA) overnight at 4 °C. On the second day, samples were incubated with 1:200 donkey anti-rabbit IgG conjugated with either tetramethylrhodamine (TRITC) or fluorescein isothiocyanate (FITC) for 1 h. After washing, the samples were incubated with the TUNEL reaction mixture (Fluorescein or TMR Red) for 1 h at 37 °C in the dark. Nuclei were then counterstained with DAPI, and apoptosis was evaluated by counting the number of TUNEL ^+^ CM nuclei relative to the total number of CM nuclei in each field ^8, 9^.

***Western Blot***: to determine which signaling pathways were involved in the cytoprotection of hiPSC-CMs after treatment with ^B2MKO^hiPSC-NVs, three key pathways, including ERK1/2, p38MAPK, and AKT, were assessed. hiPSC-CMs treated with DPBS or 5 × 10^9 B2MKO^hiPSC-NVs/mL were harvested at various time points (0, 0.5, 1.5, 3, 6, and 24 h), and total proteins were isolated using M-PER Mammalian Protein Extraction Reagent (78501, Thermo Scientific) [9,35]. Proteins were separated on a 10 % Mini-PROTEAN TGX Precast Gel (4561036, Bio-Rad), transferred to nitrocellulose membrane, and blocked with 5 % non-fat milk in TBST. Blots were incubated overnight at 4°C with primary antibodies (Supplemental Table 4): rabbit anti-phosphorylated ERK1/2, ERK1/2, phosphorylated P38MAPK, P38MAPK, phosphorylated AKT (pAKT), AKT, and GAPDH. The next day, goat anti-rabbit IgG conjugated with HRP was used to detect the binding of antibodies. The binding of antibodies was visualized using the SuperSignal West Femto Maximum Sensitivity Substrate and captured using ChemiDoc™ MP System. Protein expression levels were normalized to GAPDH and expressed as percentages of GAPDH.

***Inhibitor treatment:*** to assess whether blocking the upregulated pathway could inhibit the cytoprotective effect by ^B2MKO^hiPSC-NVs, hiPSC-CMs were pre-treated 5 μM MK-2206 (HY-108232, MedChem Express), an AKT inhibitor, for 30 min before the addition of 5 × 10^9 B2MKO^hiPSC-NVs/mL. Cells were then cultured under hypoxic conditions for 24 h. The supernatants were collected to measure LDH intensity, and apoptotic hiPSC-CMs were visualized using an In situ Cell Death Detection Kit, as described above.

### 2.7 H_2_O_2_ scavenging effect of ^B2MKO^hiPSC-NVs

To assess whether ^B2MKO^hiPSC-NVs can protect hiPSC-CMs from ROS-induced injury, HBSS containing 200 μM H_2_O_2_ was mixed with 6.25 × 10^7^ to 10 × 10^9 B2MKO^hiPSC-NVs/mL or without NVs for 10 min at room temperature. The mixtures were then used to culture hiPSC-CMs for 30 min in an incubator at 37°C. The supernatants were collected to determine the LDH intensity as described ^8, 9^.

After incubation with 6.25 × 10^7^ to 10 × 10^9 B2MKO^hiPSC-NVs/mL for 10 min, the concentrations of H_2_O_2_ in HBSS, which originally contained 200 μM H_2_O_2_, were measured using the Hydrogen Peroxide Assay Kit (ab138886, Abcam).

### 2.8 Differentiation, purification, and characterization of ^B2MKO^hiPSC-CMs

hiPSCs were differentiated into hiPSC-CMs as described previously [5,29]. hiPSC-CMs were purified for 6 days as described previously [29,36,37].

***Interferon-γ (IFNγ) stimulation*:**^WT^hiPSC-CMs and ^B2MKO^hiPSC-CMs were cultured in RPMI medium supplemented with a 100x dilution of B27 (B27/RPMI) with or without 25 ng/mL IFNγ for 48 h. Proteins were extracted with PhosphoSafe™ Extraction Reagent (Merck, Germany) for Western Blot [8,9,38]. Briefly, proteins were separated on an SDS-polyacrylamide gel, transferred onto nitrocellulose membranes, and blocked with 5 % non-fat milk in TBST. The membranes were then incubated with rabbit anti-B2M or rabbit anti-Class II transactivator (CIITA) (Supplemental Table 4) primary antibodies at 4°C overnight. Goat anti-rabbit IgG conjugated with HRP was used to detect the binding of antibodies. Imaging the specifically targeted proteins was performed as described above.

### 2.9 Proliferation of cultured hiPSC-CMs

***Immunofluorescence staining:*** hiPSC-CMs (1.5 × 10^4^ cells/well) were seeded into the wells of 4-well chamber slides and cultured in 1 mL B27/RPMI medium with or without 2.5 × 10^9^, 5 × 10^9^, or 10 × 10^9 B2MKO^hiPSC-NVs/mL for 48 h. The cells were fixed, permeabilized, blocked, and incubated with 10 % PBS containing either a 1:200 dilution of rabbit anti-phospho-Histone H3 (Ser10) (pH3, 06–570, Millipore-Sigma, USA) or a 1: 50 dilution of rabbit anti-Aurora B kinase (ABK, ab2254, Abcam, USA) at 4°C overnight. On the second day, donkey anti-rabbit IgG conjugated with FITC was applied to visualize pH3 or ABK protein expression. hiPSC-CMs were visualized using a 1:20 dilution of mouse anti-cardiac troponin T (cTnT, 564767, BD) antibody conjugated with phycoerythrin (PE). hiPSC-CMs (i.e., cTnT-positive cells) that expressed pH3 or ABK with disassembled sarcomeres and cleavage furrows were counted and reported as a fraction of 10,000 hiPSC-CMs [3,8].

***Cell number count***: The proliferation of hiPSC-CMs was evaluated by culturing the cells with or without 5 × 10^9 B2MKO^hiPSC-NVs/mL in 6-well plates (2 × 10^5^ cells/well in 2 mL B27/RPMI). The medium was changed every 2 days for 6 days, after which the cells were counted. The results were expressed as percentages after normalized to the starting cell number (2 × 10^5^ cells/well). Additionally, on day 6, 100 μL of CCK-8 buffer was added to each well, and the cells were incubated at 37 °C for 4 h. Supernatants were collected, and absorbance was measured at 450 nm using a TECAN microplate reader. The results were presented as percentages, normalized to the measurement from the supernatant without ^B2MKO^hiPSC-NV treatment, which was considered as 100 %.

***Flow cytometry for cell cycle analysis***: for cell cycle analysis, 5 × 10^5^ hiPSC-CMs were cultured in B27/RPMI supplemented with 5 × 10^9^ or 10 × 10^9 B2MKO^hiPSC-NVs/mL or without ^B2MKO^hiPSC-NVs in 6-well plates. The medium was changed every 2 days for 6 days. On day 6, cells were harvested, fixed with ice-cold 70 % ethanol for at least 30 min, and stained with a staining-buffer containing 0.1 % Triton X-100, 200 μg/mL RNase A, 20 μg/mL propidium iodide in PBS for 30 min at room temperature. Cell cycles were analyzed using a BD LSR Fortessa flow cytometer.

***Western Blot analysis*:** to determine whether YAP signaling is involved in hiPSC-CM proliferation after ^B2MKO^hiPSC-NV treatment, hiPSC-CMs were seeded into 12-well plates and treated with DPBS or 5 × 10^9^/mL ^B2MKO^hiPSC-NVs. Cells were then harvested at 0, 0.5, 1.5, 3, 6 and 24 h after treatment using M-PER Mammalian Extraction Reagent. Briefly, proteins were separated on a 10 % Mini-PROTEAN TGX Precast Gel and transferred onto a nitrocellulose membrane. The blots were incubated with primary antibodies (Supplemental Table 4): rabbit anti-phosphorylated YAP (ser127) (pYAP), YAP, or GAPDH, at 4°C overnight. Goat anti-rabbit IgG conjugated with HRP was used to detect the binding of antibodies. The imaging of the specifically targeted proteins was described above.

***Inhibitor treatment***: to further determine whether YAP signaling is responsible for ^B2MKO^hiPSC-NV-induced hiPSC-CM proliferation, cells were pretreated with 1 μM Verteporfin (HY-B0146, MedChem Express), a YAP inhibitor, for 30 min before adding ^B2MKO^hiPSC-NVs to the cell culture medium. Twenty-four hours later, hiPSC-CMs were fixed, permeabilized, blocked, and incubated with 10 % PBS containing rabbit anti-YAP (14074S) or rabbit anti-ABK along with mouse anti-cTnT conjugated with PE at 4°C overnight. On the following day, samples were incubated with 10 % PBS containing donkey anti-rabbit IgG conjugated with FITC for 1 h at room temperature. Finally, cells were stained with DAPI, washed, and mounted in Vectashield, and visualized using a confocal laser scanning microscope. hiPSC-CMs that exhibited nuclear YAP protein expression or ABK with disassembled sarcomeres and cleavage furrows were counted and reported as a fraction of 10,000 hiPSC-CMs [8].

### 2.10 Mouse heart models of I/R and treatment

All animal procedures and protocols involved were approved by the Institutional Animal Care and Use Committee of the University of Alabama at Birmingham, USA and performed according to the guidelines of the National Institutes of Health (NIH publication No 85-23). Mice were housed at 25 °C with a 12 h light–dark cycle and had free access to food and water. Cardiac I/R injury was induced in C57BL/6 mice (25–30 g, 12–14 weeks of age, both male and female) as described previously [39]. Briefly, mice were anesthetized with 2–2.5 % isoflurane, and the left anterior coronary artery was occluded with an 8-0 suture for 1 h. The suture was then removed to allow reperfusion. Mice were randomly assigned to two groups: 30 μL 0.9 % NaCl (the I/R group, n = 18) or 30 μL 0.9 % NaCl containing 2 × 10^10 B2MKO^hiPSC-NVs (the I/R + NV group, n = 18), using insulin syringes with 29G needles. Treatments were injected into the infarcted left ventricular (LV) anterior wall. A third group of age-matched animals (the Sham group, n = 14) underwent open-chest surgery without arterial ligation or subsequent treatment. Buprenorphine (0.01–0.02 mg/kg, *i.m*.) and Carprofen (5 mg/kg, *s.c.*) were administered before surgery and for 3 days after surgery. Euthanasia was performed in anesthetized animals (2–2.5 % isoflurane) via direct intracardial injection of 100 mg/mL/Kg KCl.

### 2.11 Echocardiography

Transthoracic echocardiography was conducted using a Vevo 2100 echocardiographic system (VisualSonics, VSI, Toronto, Canada) with an MS400 transducer as described [9]. All measurements were carried out in a blinded manner. Mice were anesthetized with isoflurane (1–2 %), and their chests were shaved. The animals were then positioned in a dorsal decubitus position. B-mode parasternal long axis images were collected, ensuring well-visualized aortic valves and the apex at maximum dimension for 10 cardiac cycles, and stored for offline analysis [9,40]. The endocardium of the LV was traced manually at peak systole and peak diastole to measure left ventricular end diastolic volume (LVEDV) and left ventricular end systolic volume (LVESV). LV ejection fraction (LVEF) was then calculated using the following formula: LVEF = (LVEDV-LVESV)/LVEDV × 100 %.

### 2.12 Histological and immunochemical assessments

***Apoptosis (TUNEL assay)*:** Cryosections of cardiac tissue were evaluated using an In-situ Cell-death Detection Kit, as described previously [9,41]. Sections were stained with rabbit anti-cTnI (ab47003, Abcam) to visualize CMs. Both the total number of CM nuclei and the number of apoptotic CM nuclei were counted in each tissue section under 20 × magnification, and analysis was conducted on four animal hearts per group [9,41].

***Infarct size and LV wall thickness measurements:*** heart tissue sections were paraffin-embedded and stained using an Accustain Trichrome Stains (Masson) kit (Sigma-Aldrich, USA) to differentiate healthy myocardium from fibrotic tissue. Infarct size was calculated as the ratio of the length of the fibrous scar to the circumference of the LV free wall [9]. Measurements were conducted at the mid-injury site in nine animals per group.

***Mouse CM proliferation:*** Paraffin-embedded heart sections were stained with primary mouse IgM anti-α-sarcomeric actin antibodies (α-SA, A2172, MilliporeSigma) and primary rabbit anti-pH3 (06–570, MilliporeSigma) or rabbit anti-ABK (ab2254, Abcam) antibodies. Primary antibodies were visualized with donkey anti-rabbit IgG conjugated with FITC, and the sections were imaged using a FV3000 confocal laser scanning microscope (Olympus). Total CMs, pH3-positive CMs, and ABK-positive CMs were counted in 5 animals per group at week 1 and in 9 animals per group at week 4. A total of 6–7 slides per animal and 2-3 sections per slide were analyzed.

### 2.13 Statistical analysis

Results were presented as mean ± standard deviation (SD), and statistical analyses were performed using SPSS (version 28.0) software. Differences between two groups were assessed using two-tailed unpaired T-Tests, and overall differences among groups were analyzed using one-way analysis of variance (ANOVA). When significant differences were identified by ANOVA, post-hoc analysis was performed with the Tukey test. To identify proteins that were significantly more abundant in ^B2MKO^hiPSC-NVs than in ^B2MKO^hiPSCs, raw protein abundance data were log10-transformed and analyzed using a two-tailed unpaired T-Test, followed by Benjamini-Hochberg correction. The P value of <0.05 was considered statistically significant.

## 3 Results

### 3.1 ^B2MKO^hiPSC-NVs are nano-sized vesicles and internalized by cultured hiPSC-CMs

Two ^B2MKO^hiPSC lines were generated using CRISPR/Cas9 genome editing to delete 198-bp or 209-bp from human β2-microglobulin exon 2 (NM_004048.4) (Supplemental Fig. 1). Four clones (C39, C59, C64, and C66) were obtained with the 198-bp deletion, and one clone (C55) was obtained with the 209-bp deletion. All subsequent experiments were conducted with ^B2MKO^hiPSCs from clone C66. Flow cytometry analyses (Supplemental Fig. 2) confirmed that B2M, HLA-A/B/C, and HLA-DR/DQ/DP proteins were nearly undetectable in ^B2MKO^hiPSCs, while HLA-E and HLA-G proteins were significantly less abundant in ^B2MKO^hiPSCs compared to ^WT^hiPSCs. Approximately 84 % of both ^WT^hiPSCs and ^B2MKO^hiPSCs expressed CD47, which inhibits natural killer cells [42]. The ^B2MKO^hiPSCs formed colonies in culture, expressed the pluripotency markers OCT3/4 and SSEA4, and formed teratomas containing mesodermal (muscle), ectodermal (epidermis), and endodermal (gastrointestinal gland) cells. Karyotype analysis appeared normal (Supplemental Fig. 3). When hiPSCs were differentiated into hiPSC-CMs and treated with INFγ, B2M protein expression increased significantly in ^WT^hiPSC-CMs, but not in ^B2MKO^hiPSC-CMs (Supplemental Fig. 4A and B). The expression of CIITA remained unchanged in both hiPSC-CM populations (Supplemental Fig. 4A and C).

**Fig. 1:**
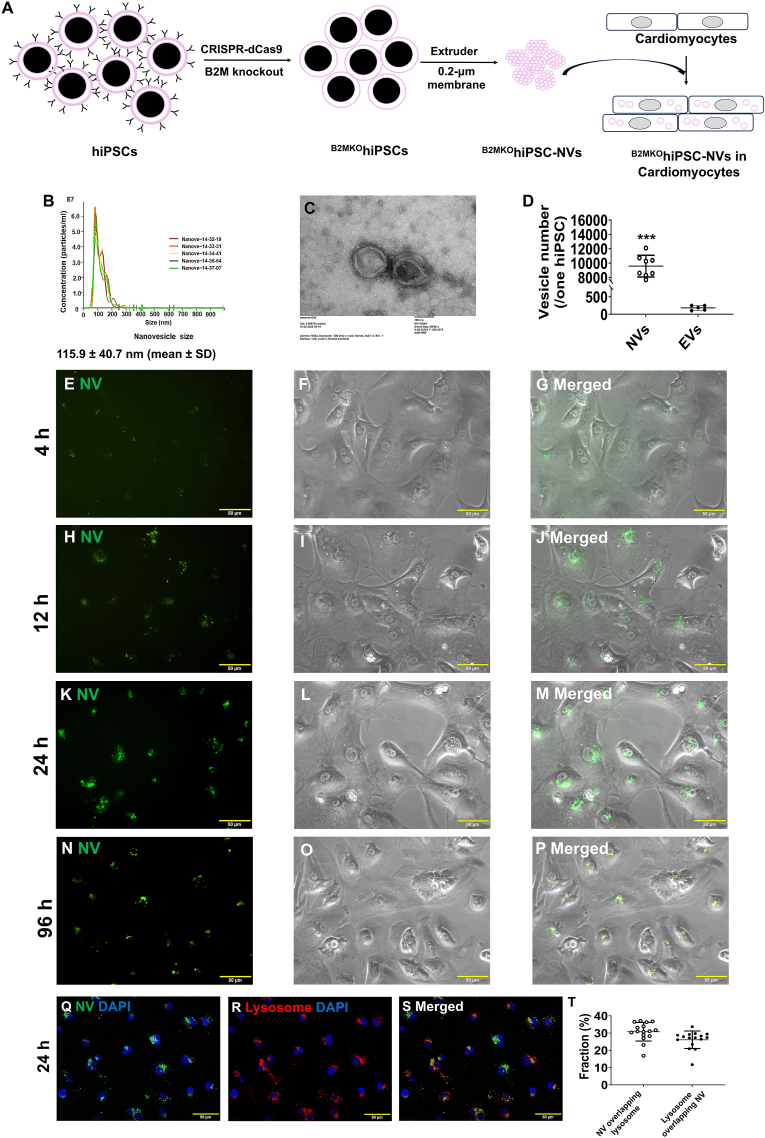
**Characterization of ^B2MKO^hiPSC-NVs. (A)** Schematic illustration of the manufacturing of ^B2MKO^hiPSC-NVs for cardiomyocyte protection and regeneration. **(B)** hiPSC-NV size was measured via nanoparticle tracking analysis. **(C)** hiPSC-NV morphology was evaluated via a transmission electron microscopy. **(D)** The number of NVs or extracellular vesicles (EVs) generated per one ^B2MKO^hiPSC was calculated (n = 8 or 5 biological replicates). **(E**–**P)** hiPSC-CMs were incubated with PKH67-labeled ^B2MKO^hiPSC-NVs for 4 h **(E**–**G)**, 12 h **(H**–**J)**, 24 h **(K**–**M)**, and 96 h **(N**–**P)**. ^B2MKO^hiPSC-NVs were visualized by green fluorescence **(E, H, K, and N)**, hiPSC-CMs were visualized via phase-contrast **(F, I, L and O)**, and the two images were merged to identify ^B2MKO^hiPSC-NVs that were internalized by the cells **(G, J, M, and P)**. hiPSC-CMs were incubated with PKH67-labeled ^B2MKO^hiPSC-NVs for 24 h, and lysosomes were labeled with LysoView™ 550. **(Q)**^B2MKO^hiPSC-NVs were visualized via green fluorescence, **(R)** hiPSC-CM lysosomes were visualized via red fluorescence, and **(S)** the merged images were used to determine the overlap between green and red fluorescences. **(T)** Quantification of the fractions (percentages) of green fluorescence (^B2MKO^hiPSC-NVs) overlapping red fluorescence (lysosome) and red fluorescence overlapping green fluorescence, respectively, using image J. Values were presented as mean ± SD.Independent *t*-test: ∗∗∗p < 0.001.

The manufacturing process of ^B2MKO^hiPSC-NVs from ^B2MKO^hiPSCs was illustrated in Fig. 1A. The ^B2MKO^hiPSC-NVs had diameters of approximately 115.9 ± 40.7 nm with bilayer-lipid membranes (Fig. 1B and C), and the zeta potential was −13.29 ± 2.15 mv. Using an extruder, approximately 9500 NVs could be manufactured from a single hiPSC, which was about 52 times the number of EVs (approximately 184) produced by one hiPSC every 24 h (Fig. 1D),

When ^B2MKO^hiPSC-NVs labeled with PKH67 were cultured with hiPSC-CMs, green fluorescence was observed in the hiPSC-CMs starting 4 h after co-culture (Fig. 1E–G). The intensity of the green fluorescence continued to increase in the hiPSC-CMs after 12 and 24 h (Fig. 1H–M), confirming that ^B2MKO^hiPSC-NVs were internalized by the cells. Green fluorescence remained visible in the hiPSC-CMs up to day 4 after co-culture with PKH67-labeled ^B2MKO^hiPSC-NVs (Fig. 1N–P), indicating that intact ^B2MKO^hiPSC-NVs were persisted within the cells for at least 4 days.

Furthermore, when lysosomes in hiPSC-CMs were labeled with a red-fluorescent dye and the cells were cultured with PKH67-labeled ^B2MKO^hiPSC-NVs (Fig. 1Q–S), less than 40 % of the green and red fluorescence overlapped (Fig. 1T). This suggested that most of the internalized ^B2MKO^hiPSC-NVs did not directly undergo lysosomal degradation.

When ^B2MKO^hiPSC-NVs and ^B2MKO^hiPSCs were injected into the flanks of NOD-SCID mice, teratomas were only observed in the right flanks that received the ^B2MKO^hiPSC injection 1.5 months after treatment (Supplemental Fig. 5). No tumors were observed when the mice were euthanized at 2 months after the ^B2MKO^hiPSC-NV injection.

### 3.2 **B2MKO reduced the immunogenicity of**^B2MKO^**hiPSC-NVs**

After culturing human CD8^+^ T cells with either 0.9 % NaCl, ^WT^hiPSC-NVs, or ^B2MKO^hiPSC-NVs, the proportions of CD69^+^/CD8^+^, CD38^+^/CD69^+^/CD8^+^, and total CD38^+^/CD8^+^ plus CD69^+^/CD8^+^ T cells were the highest in the ^WT^hiPSC-NVs group, which were significantly higher than those in the 0.9 % NaCl or ^B2MKO^hiPSC-NVs treated CD8^+^ T cells (Supplemental Fig. 6A). No significant differences were observed between the 0.9 % NaCl and ^B2MKO^hiPSC-NV-treated CD8^+^ T cell profiles.

Furthermore, when 0.9 % NaCl, or ^WT^hiPSC-NVs, or ^B2MKO^hiPSC-NVs were intraperitoneally injected into C57BL/6 mice, CD4, CD8, CD69, and CD8/CD69-positive lymphocytes were similarly abundant in all three treatment groups on Day 1 (Supplemental Fig. 6B). However, by Day 3, CD8^+^ T cells were significantly more frequently found in animals treated with ^WT^hiPSC-NVs than those treated with 0.9 % NaCl or ^B2MKO^hiPSC-NVs. The CD8^+^ T cell measurements in the 0.9 % NaCl and ^B2MKO^hiPSC-NV groups were similar. Interestingly, both ^WT^hiPSC-NV and ^B2MKO^hiPSC-NV administrations were associated with significant declines in CD69^+^ T lymphocytes (Supplemental Fig. 6C).

### 3.3 Proteomic characteristics of ^B2MKO^hiPSCs and ^B2MKO^hiPSC-NVs

**Fig. 2:**
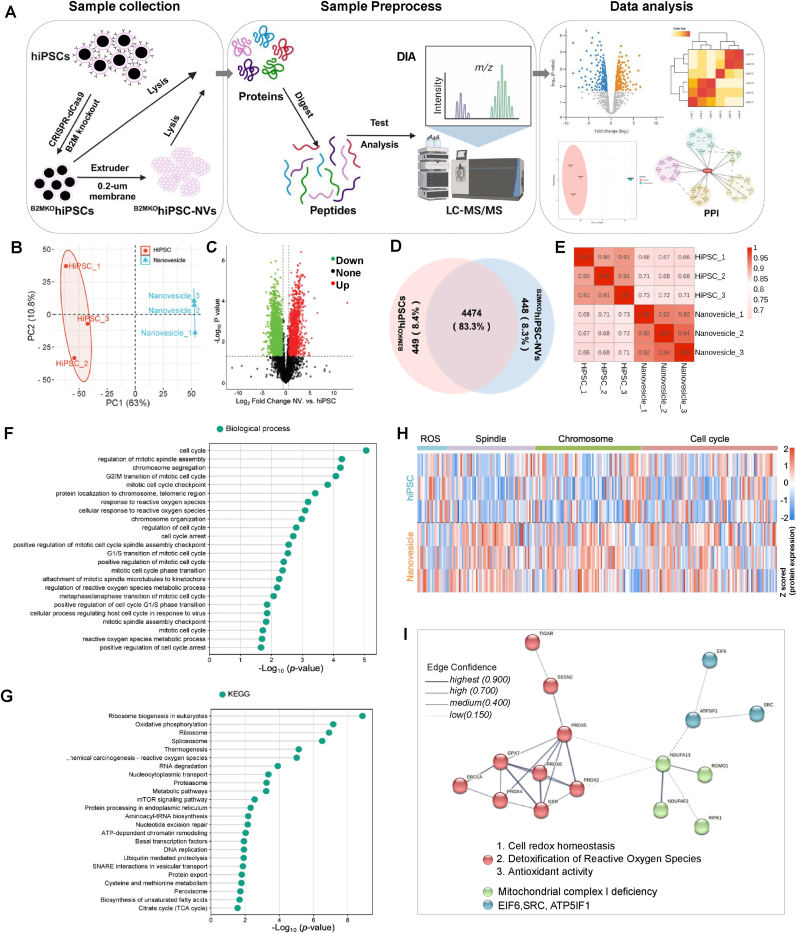
**Proteomic analyses revealed that ^B2MKO^hiPSC-NVs contained abundant proteins involved in biological processes related to ROS, the mitotic spindle, chromosomes, and the cell cycle.** Proteomic analyses were performed on ^B2MKO^hiPSCs and ^B2MKO^hiPSC-NVs. **(A)** Schematic representation of data-independent acquisition (DIA) quantitative proteomics and data analysis. **(B)** Principal component analysis (PCA) for the detected proteins in ^B2MKO^hiPSCs and ^B2MKO^hiPSC-NVs. **(C)** Volcano plots comparing protein levels in ^B2MKO^hiPSCs and ^B2MKO^hiPSC-NVs, with significantly upregulated proteins shown in red and downregulated proteins shown in green. **(D)** Venn diagram illustrating the number of proteins expressed in ^B2MKO^hiPSCs and ^B2MKO^hiPSC-NVs. **(E)** Spearman correlation analysis of co-expressed proteins in ^B2MKO^hiPSCs and ^B2MKO^hiPSC-NVs. **(F)** Biological processes (BP) enrichment analysis of co-expressed proteins in ^B2MKO^hiPSCs and ^B2MKO^hiPSC-NVs, with GO terms related to ROS, the mitotic spindle, chromosomes, and the cell cycle. **(G)** Kyoto Encyclopedia of Genes and Genomes (KEGG) pathway analysis for co-expressed proteins in ^B2MKO^hiPSCs and ^B2MKO^hiPSC-NVs. **(H)** Heatmap illustrating the magnitudes of co-expressed proteins related to ROS, the mitotic spindle, chromosomes, and the cell cycle in ^B2MKO^hiPSCs and ^B2MKO^hiPSC-NVs. **(I)** Protein-protein interaction network for proteins related to ROS, based on three independent analyses of ^B2MKO^hiPSCs and ^B2MKO^hiPSC-NVs. (n = 3 biological replicates).

A label-free proteomic analysis was conducted to systematically investigate the molecular alterations between ^B2MKO^hiPSCs and ^B2MKO^hiPSC-NVs. Both ^B2MKO^hiPSCs (n = 3) and ^B2MKO^hiPSC-NVs (n = 3) were subjected to protein extraction, peptide digestion, and liquid chromatography-mass spectrometry/mass spectrometry (LC-MS/MS) detection analysis (Fig. 2A). Principal component analysis demonstrated clear separation between the clusters of ^B2MKO^hiPSCs and ^B2MKO^hiPSC-NVs (Fig. 2B). The volcano plot highlighted significant differences in protein expression between the two groups (Fig. 2C). Peptides and proteins with an FDR not exceeding 1.0 % were retained. In total, 4923 were identified in ^B2MKO^hiPSCs, and 4922 proteins were identified in ^B2MKO^hiPSC-NVs, with 4474 proteins co-expressed in both (Fig. 2D, Supplemental Fig. 7). Among these, 283 membrane proteins, 872 nuclear proteins, and 890 cytoplasmic proteins were identified in ^B2MKO^hiPSC-NVs. Correlation analysis of co-expressed proteins (Fig. 2E) and all identified proteins (Supplemental Fig. 7C) showed correlations were greater than or equal to 0.90, indicating the high quality of the mass spectrometry analysis. Further analysis focused on the co-expressed proteins to explore those related to cardioprotection and cell proliferation in ^B2MKO^hiPSC-NVs. The GO enrichment analysis revealed that significant enrichment was found in biological processes associated with reactive oxygen species (ROS) metabolism, cellular responses to ROS, chromosome dynamics, spindle function, and the cell cycle in ^B2MKO^hiPSC-NVs (Fig. 2F, Supplemental Fig. 8). Additionally, KEGG pathway enrichment analysis showed significant enrichment in signaling pathways related to cellular responses to stimuli, DNA repair, RNA metabolism, gene expression, autophagy, and cell cycle regulation (Fig. 2G, Supplemental Figs. 9 and 10).

A heatmap illustrating the magnitude of co-expressed proteins related to ROS, the mitotic spindle, chromosomes, and the cell cycle in ^B2MKO^hiPSCs and ^B2MKO^hiPSC-NVs was shown in Fig. 2H. A total of 20 ROS-related proteins, 59 mitotic spindle proteins, 93 chromosomes-related proteins, and 121 cell cycle-related proteins were co-expressed in both ^B2MKO^hiPSCs and ^B2MKO^hiPSC-NVs (Supplemental Figs. 11–14). Additionally, quantitative analysis (fold change >1.5) confirmed the enrichment of specific proteins in ^B2MKO^hiPSC-NVs: 4 ROS scavengers (NUDT2, PRDX2, PRDX6, and GSR) (Supplemental Fig. 11), 32 spindle-related proteins (Supplemental Fig. 12), 46 chromosome-associated proteins (Supplemental Fig. 13), and 55 cell cycle proteins (Supplemental Fig. 14).

Further statistical analysis using an unpaired T-Test followed by Benjamini-Hochberg correction revealed that 4 ROS-related proteins, 16 mitotic spindle proteins, 17 chromosome-associated proteins, and 24 cell cycle-related proteins were significantly more abundant in ^B2MKO^hiPSC-NVs compared to ^B2MKO^hiPSCs (Supplemental Figs. 11–14). Notably, the inner centromere protein (INCENP), which participates in cytokinesis, and the proliferation marker Ki-67 (MKI67), which regulates mitotic nuclear division, were 37 % and 887.6 % more abundant, respectively, in ^B2MKO^hiPSC-NVs than in ^B2MKO^hiPSCs. Western blot analysis of 11 proteins involved in anti-ROS (i.e. PRDX2, PRDX6, and GSR), spindle (i.e. TPR, SKA2, RIF1, INCENP), chromosome (i.e. SKA2, RIF1, Ki67, CBX5, and INCENP), and the cell cycle (i.e. RBBP4/7, CKS1/2, RIF1, and TPR) confirmed that these proteins were significantly more abundant in ^B2MKO^hiPSC-NVs than in ^B2MKO^hiPSCs (Supplemental Fig. 15). Thus, while the protein cargo of ^B2MKO^hiPSC-NVs shared similarities with the protein expression profile of ^B2MKO^hiPSCs, ^B2MKO^hiPSC-NVs appear to be enriched in proteins associated with anti-ROS activity, the mitotic spindles, chromosome, and cell cycle regulation.

To further elucidate the functional interactions of the enriched proteins, we conducted a PPI network analysis of the co-expressed proteins. The network analysis revealed strong interactions among ROS-related, spindle-associated, chromosome-related, and cell cycle-related proteins. This suggested that ^B2MKO^hiPSC-NVs exerted cardioprotective functions through multiple signaling pathways (Fig. 2I, Supplemental Fig. 16).

### 3.4 ^B2MKO^hiPSC-NVs protected hiPSC-CMs from hypoxia or H_2_O_2_-induced injury

**Fig. 3:**
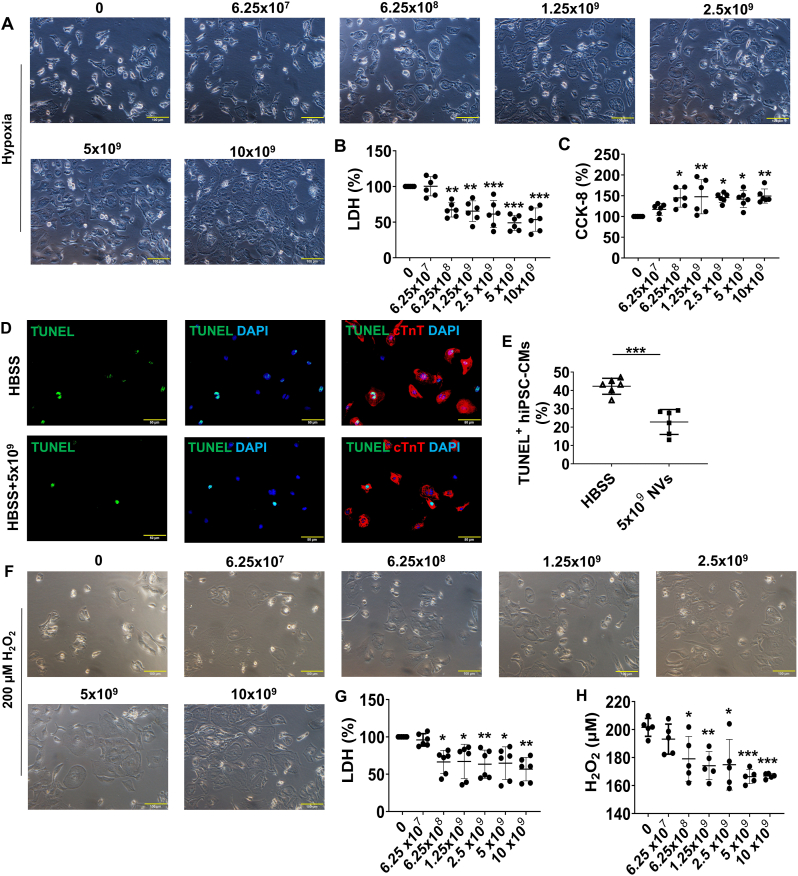
**^B2MKO^hiPSC-NVs improved ^B2MKO^hiPSC-CM survival under hypoxia and in the presence of H_2_O_2_. (A)**^B2MKO^hiPSC-CMs were cultured under hypoxia in the Hanks' balance salt solution (HBSS) supplemented with the indicated concentrations of ^B2MKO^hiPSC-NVs for 24 h. **(B)** Medium LDH levels were measured, normalized to the measurements in the absence of ^B2MKO^hiPSC-NVs, and presented as a percentage (n = 6 biological replicates). **(C)** CCK-8 assay was performed to measure the viability of ^B2MKO^hiPSC-CMs cultured under hypoxia in HBSS alone or in HBSS with the indicated concentrations of ^B2MKO^hiPSC-NVs for 24 h. The results were normalized to the measurements in the absence of ^B2MKO^hiPSC-NVs and presented as a percentage (n = 6 biological replicates). **(D)** Representative images of TUNEL-stained ^B2MKO^hiPSC-CMs cultured under hypoxia in HBSS alone or HBSS + 5 × 10^9 B2MKO^hiPSC-NVs/mL for 24 h. **(E)** The proportion of cTnT^+ B2MKO^hiPSC-CMs that were positive for TUNEL was calculated and presented as a percentage (n = 6 biological replicates). **(F)**^B2MKO^hiPSC-CMs were cultured with 200 μM H_2_O_2_, which was premixed with the indicated concentrations of ^B2MKO^hiPSC-NVs, for 30 min. **(G)** Medium LDH levels were measured, normalized to the measurements in the medium with 200 μM H_2_O_2_ in the absence of ^B2MKO^hiPSC-NVs, and presented as a percentage (n = 6 biological replicates). **(H)** The concentrations of H_2_O_2_ in HBSS after incubation with the indicated concentrations of ^B2MKO^hiPSC-NVs were measured (n = 5 biological replicates). Values are presented as mean ± SD. Panels B, C, G, and H: one-way ANOVA followed by the Tukey test and panel D: independent T-Test; ∗*P* < 0.05, ∗∗*P* < 0.01, and ∗∗∗*P* < 0.001.

Given that the cargos of ^B2MKO^hiPSC-NVs contained a higher abundance of proteins involved in cardioprotection, including ROS scavengers, we assessed whether ^B2MKO^hiPSC-NVs could protect ^B2MKO^hiPSC-CMs from hypoxia or H_2_O_2_ induced injury. When ^B2MKO^hiPSC-CMs were cultured under hypoxic conditions, the LDH concentration in the supernatant was significantly lower in cells cultured with ^B2MKO^hiPSC-NVs, starting at a concentration of 6.25 × 10^8^ NVs/mL, compared to those without ^B2MKO^hiPSC-NVs (Fig. 3A and B). The CCK-8 assay further confirmed that a higher number of viable ^B2MKO^hiPSC-CMs survived after treatment with ^B2MKO^hiPSC-NVs, beginning at 6.25 × 10^8^ NVs/mL (Fig. 3C). Additionally, treatment with 5 × 10^9 B2MKO^hiPSC-NVs/mL significantly reduced the proportion of TUNEL-positive hiPSC-CMs under hypoxic conditions (Fig. 3D and E).

When 200 μM H_2_O_2_ was pre-incubated with ^B2MKO^hiPSC-NVs for 10 min and then applied to hiPSC-CMs for 30 min, the level of LDH in the supernatant was significantly lower in hiPSC-CMs co-cultured with H_2_O_2_ and ^B2MKO^hiPSC-NVs, starting at a concentration of 6.25 × 10^8^ NVs/mL, compared to those treated with H_2_O_2_ alone (Fig. 3F and G). The concentrations of H_2_O_2_ in HBSS significantly decreased after incubation with ^B2MKO^hiPSC-NVs, starting from 6.25 × 10^8^ NVs/mL concentration (Fig. 3H).

Additionally, cryo-preserved ^B2MKO^hiPSC-NVs, starting from 6.25 × 10^8^ NVs/mL, significantly reduced LDH abundance in cells compared to those without ^B2MKO^hiPSC-NVs (Supplemental Fig. 17A and B). However, a higher concentration of cryo-preserved ^B2MKO^hiPSC-NVs (2.5 × 10^9 B2MKO^hiPSC-NVs/mL) was required to protect hiPSC-CMs from H_2_O_2_-induced damage (Supplemental Fig. 17C and D). These data suggest that both freshly prepared and cryo-preserved ^B2MKO^hiPSC-NVs protected cultured hiPSC-CMs from cytotoxicity associated with hypoxia and H_2_O_2_, as well as from hypoxia-induced apoptosis.

### 3.5 ^B2MKO^hiPSC-NVs protected hiPSC-CMs from hypoxia through AKT signaling pathway

**Fig. 4:**
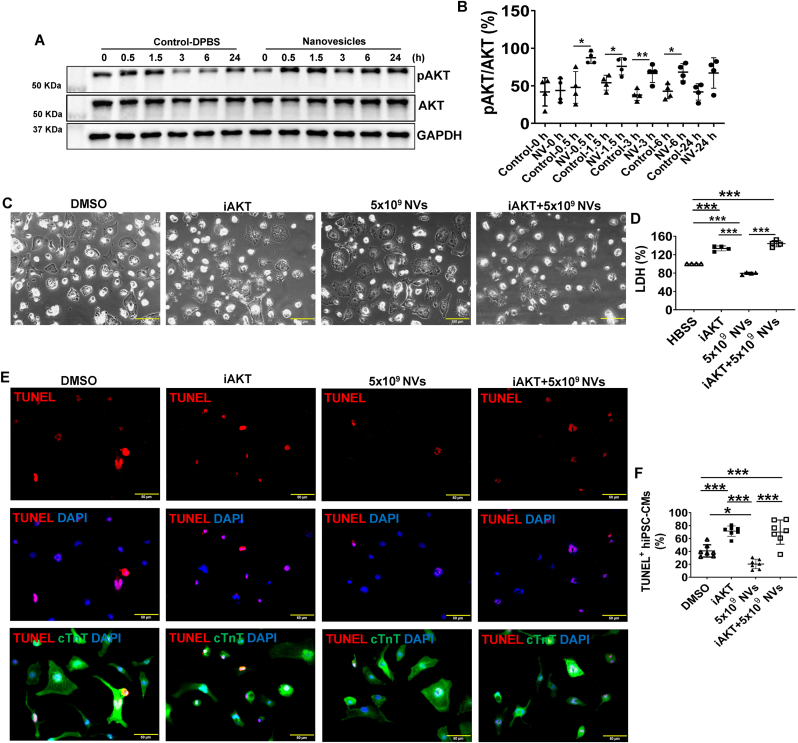
**AKT signaling pathway is involved in the cytoprotection of ^B2MKO^hiPSC-NVs from hypoxia. (A)** Representative Western Blot images of hiPSC-CMs treated with or without 5 × 10^9 B2MKO^hiPSC-NVs for protein expressions of pAKT and AKT. **(B)** Quantification of pAKT/AKT ratios (n = 4 biological replicates). Protein expression level of GAPDH was used as an internal control. **(C)** Representative images of hiPSC-CMs cultured in HBSS supplemented with DMSO, AKT inhibitor (iAKT), 5 × 10^9 B2MKO^hiPSC-NVs/mL, or iAKT + 5 × 10^9 B2MKO^hiPSC-NVs/mL under hypoxic conditions for 24 h. **(D)** Supernatants were collected to assess LDH levels in supernatants after iAKT, 5 × 10^9 B2MKO^hiPSC-NVs, or iAKT + 5 × 10^9 B2MKO^hiPSC-NVs treatment, and LDH was expressed as a percentage after normalized to the values from DMSO only, which were considered 100 % (n = 4 biological replicates). **(E)** Representative TUNEL-stained images of hiPSC-CMs cultured in HBSS supplemented with DMSO, iAKT, 5 × 10^9 B2MKO^hiPSC-NVs/mL, or iAKT + 5 × 10^9 B2MKO^hiPSC-NVs/mL under hypoxia for 24 h. **(F)** Quantification of TUNEL^+^ hiPSC-CMs (n = 7 biological replicates). Values are presented as mean ± SD. Panel B: independent T-Test at each time point and panels D & F: one-way ANOVA followed by the Tukey test; ∗*P* < 0.05, ∗∗*P* < 0.01, and ∗∗∗*P* < 0.001.

Western Blot analysis showed that only the AKT signaling pathway was significantly upregulated at 0.5, 1.5, 3, and 6 h after co-culturing hiPSC-CMs with ^B2MKO^hiPSC-NVs (Fig. 4A and B). When hiPSC-CMs were pre-treated with MK-2206, an AKT inhibitor (iAKT), the cytoprotective effect of ^B2MKO^hiPSC-NVs was blocked, as shown by both LDH and TUNEL assays, which indicated significant increases in hiPSC-CM injury (Fig. 4C and D) and apoptosis (Fig. 4E and F). These results indicated that ^B2MKO^hiPSC-NVs promoted hiPSC-CM viability under hypoxic conditions by targeting the AKT signaling pathway.

### 3.6 ^B2MKO^hiPSC-NVs promoted hiPSC-CM proliferation through upregulation of YAP protein expression *in vitro*

**Fig. 5:**
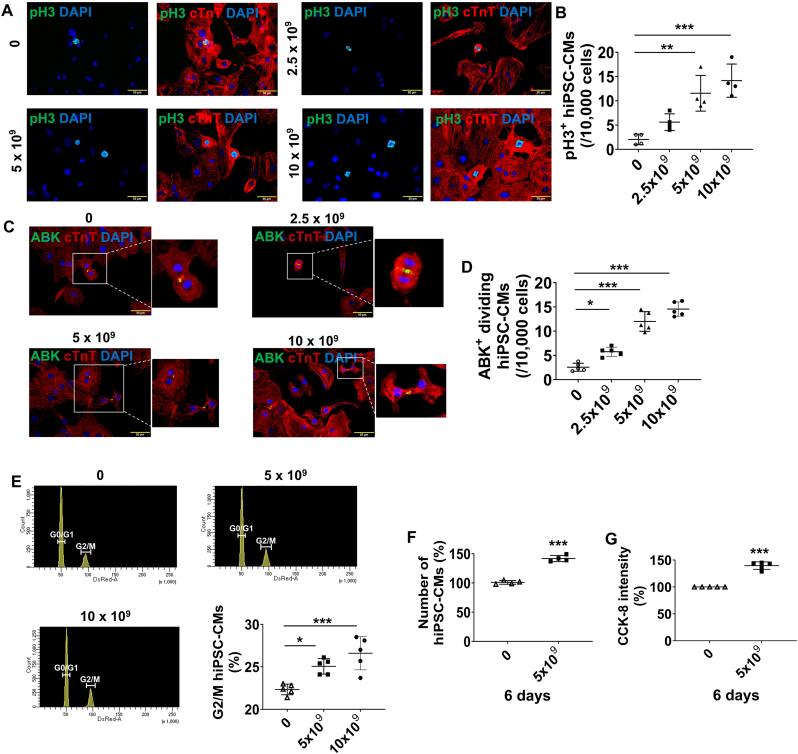
**^B2MKO^hiPSC-NVs increase proliferation of cultured hiPSC-CMs. (A)** hiPSC-CMs were treated with the indicated concentrations of ^B2MKO^hiPSC-NVs and stained for phosphorylated histone 3 (pH3), cardiac troponin T (cTnT), and DAPI by immunofluorescence. **(B)** The proportion of hiPSC-CMs that were positive for pH3 was determined (n = 4 biological replicates). **(C)** hiPSC-CMs were treated with the indicated concentrations of ^B2MKO^hiPSC-NVs and stained for the presence of Aurora B Kinase (ABK), cTnT, and DAPI by immunofluorescence. **(D)** The proportion of ABK^+^ hiPSC-CMs under cytokinesis was determined (n = 5 biological replicates). **(E)** hiPSC-CMs were cultured with the indicated concentrations of ^B2MKO^hiPSC-NVs for 6 days, then harvested, fixed, stained with propidium iodide, and analyzed for cell cycle analysis using flow cytometry. The proportion of hiPSC-CMs in G2/M phase was calculated (n = 5 biological replicates). **(F)** hiPSC-CMs were cultured with or without 5 × 10^9 B2MKO^hiPSC-NVs/mL for 6 days and then counted (n = 4 biological replicates). **(G)** CCK-8 assay was performed to measure the proliferation of hiPSC-CMs after culturing with or without 5 × 10^9 B2MKO^hiPSC-NVs/mL for 6 days. The results for hiPSC-CMs cultured with 5 × 10^9 B2MKO^hiPSC-NVs/mL were normalized to the measurements from the absence of ^B2MKO^hiPSC-NVs and presented as a percentage (n = 5 biological replicates). Values were presented as mean ± SD. Panels B, D, and E: one-way ANOVA followed by the Tukey test; panels F and G: independent T-Test; ∗*P* < 0.05, ∗∗*P* < 0.01, and ∗∗∗*P* < 0.001.

^B2MKO^hiPSC-CMs were cultured under normal oxygen conditions with serial concentrations of ^B2MKO^hiPSC-NVs. Treatment with 2.5 × 10^9^, 5 × 10^9^, or 10 × 10^9 B2MKO^hiPSC-NVs/mL dose-dependently increased the proportion of cells expressing pH3 (Fig. 5A and B) and ABK (Fig. 5C and D) by up to 6.9- and 5.7-fold, respectively. Cell cycle analysis revealed that treatment with 5 × 10^9^ or 10 × 10^9 B2MKO^hiPSC-NVs/mL significantly increased the proportion of ^B2MKO^hiPSC-CMs in the G2/M phase (Fig. 5E). Additionally, cell counts of ^B2MKO^hiPSC-CMs were 40.3 % higher after 6 days of culture with 5 × 10^9 BM2KO^hiPSC-NVs/mL compared to those cultured without ^BM2KO^hiPSC-NVs (Fig. 5F), which was further confirmed by the CCK-8 assay (Fig. 5G).

**Fig. 6:**
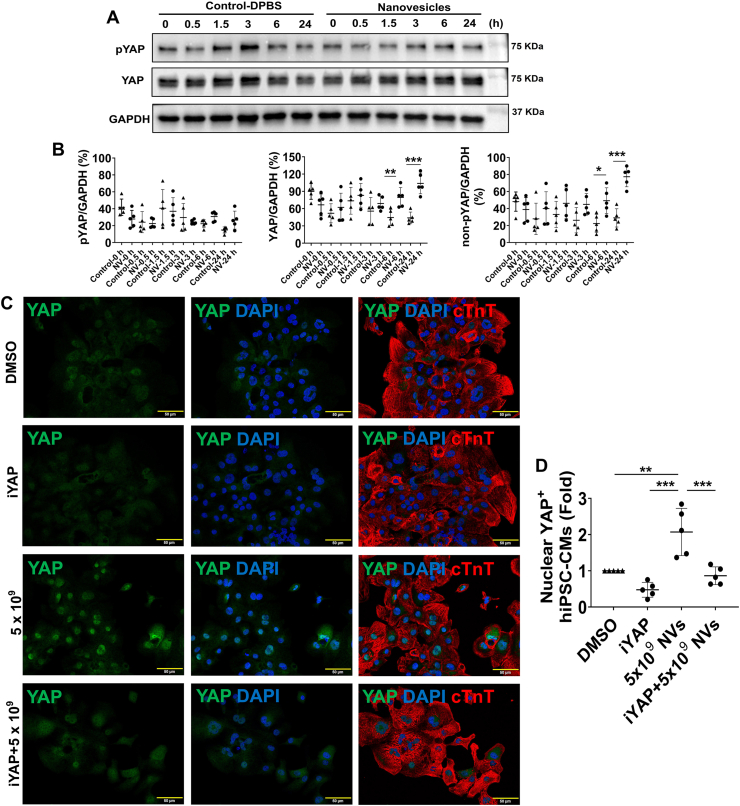
**^B2MKO^hiPSC-NVs promoted CM proliferation through the YAP signaling pathway. (A)** Representative Western Blot images of hiPSC-CMs treated with or without 5 × 10^9 B2MKO^hiPSC-NVs for the protein expression of pYAP and YAP. **(B)** Quantification of pYAP/GAPDH, YAP/GAPDH, and non-phosphorylated YAP (non-pYAP)/GAPDH ratios (n = 5 biological replicates). Protein expression levels of GAPDH were used as an internal control. **(C)** Representative images of hiPSC-CMs cultured in RPMI/B27 medium supplemented with DMSO, YAP inhibitor (iYAP), 5 × 10^9 B2MKO^hiPSC-NVs/mL, or iYAP + 5 × 10^9 B2MKO^hiPSC-NVs/mL for 24 h. Cells were then immunofluorescently stained for YAP and cardiac troponin T (cTnT) protein. **(D)** Quantification of hiPSC-CMs with YAP protein expressed in the nuclei. The number of hiPSC-CMs with nuclear YAP expression was normalized to the number in the DMSO treatment, which was considered as the baseline (100 %) (n = 5 biological replicates). **(E)** Representative images of hiPSC-CMs cultured in RPMI/B27 supplemented with DMSO, iYAP, 5 × 10^9 B2MKO^hiPSC-NVs/mL, or iYAP + 5 × 10^9 B2MKO^hiPSC-NVs/mL for 48 h. Cells were then immunofluorescently stained for aurora B kinse (ABK) and cTnT expression. **(F)** Quantification of ABK-expressing hiPSC-CMs under cytokinesis (n = 5 biological replicates). Values were presented as mean ± SD. Panel B: independent T-Test at each time point; panels D & F: one-way ANOVA followed by the Tukey test; ∗*P* < 0.05, ∗∗*P* < 0.01, and ∗∗∗*P* < 0.001.

When pYAP and YAP protein expressions were assessed via Western Blot (Fig. 6A), pYAP levels remained relatively unchanged; however, YAP protein expression was significantly upregulated at 6 and 24 h after ^BM2KO^hiPSC-NV treatment, leading to a higher proportion of non-phosphorylated YAP (non-pYAP) in hiPSC-CMs (Fig. 6B). Since non-pYAP can translocate into the cell nucleus and interact with transcriptional cofactors to activate pro-proliferative genes associated with cell proliferation [43], immunofluorescence staining was performed. This revealed that ^B2MKO^hiPSC-NV treatment significantly increased the proportion of hiPSC-CMs with abundant YAP protein in the nucleus (Fig. 6C and D). However, Verteporfin, a YAP inhibitor (iYAP), blocked YAP protein translocation into the nucleus. Additionally, hiPSC-CMs treated with Verteporfin showed a significant reduction in the number of ABK positive hiPSC-CMs undergoing cytokinesis (Fig. 6E and F). These results suggested that ^B2MKO^hiPSC-NVs target YAP signaling pathway to promote hiPSC-CM proliferation.

### 3.7 ^B2MKO^hiPSC-NVs improved recovery from myocardial injury in mice

**Fig. 7:**
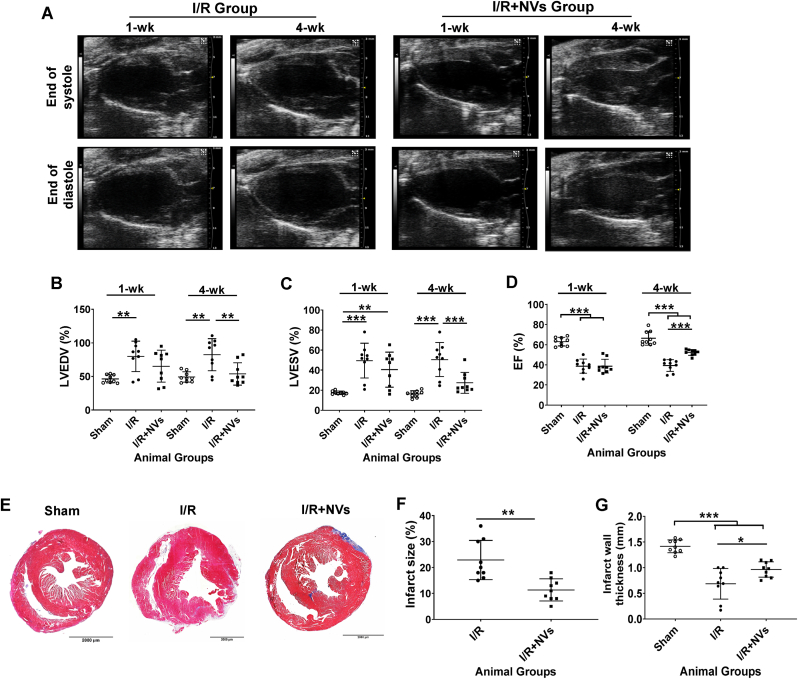
**^B2MKO^hiPSC-NV administration improved cardiac function measurements and reduced infarct size in mouse heart models of I/R injury. (A)** Representative echocardiographic images collected 1 and 4 weeks after I/R induction and treatment, showing measurements of **(B)** left ventricular end-diastolic volume (LVEDV), **(C)** left ventricular end-systolic volume (LVESV), and **(D)** ejection fraction (EF). **(E)** Sections of cardiac tissue from mice sacrificed at Week 4 were Masson-trichrome–stained. **(F)** Infarct size was calculated as the ratio of the lengths of the fibrotic region to the total circumference of the left ventricle (LV) and presented as a percentage. **(G)** The thickness of the LV wall was measured in the infarcted region of the hearts from I/R and I/R + NVs animals, as well as in the corresponding region of hearts from the Sham group. N = 9 animals in each group, and values were presented as mean ± SD. Panels B–D and G: one-way ANOVA followed by the Tukey test; panel F: unpaired T-Test; ∗*P* < 0.05, ∗∗*P* < 0.01, and ∗∗∗*P* < 0.001.

At week 1, echocardiographic analysis (Fig. 7A) showed that LVESV was significantly greater, and LVEF was significantly smaller in both the I/R and I/R + NV groups compared to the Sham group. LVEDV was also greater in the I/R and I/R + NV groups than in the Sham group, with no significant difference observed between the I/R and/R + NV groups (Fig. 7B–D). By week 4, all three parameters were significantly improved in the I/R + NV animals compared to the I/R group. Notably, no significant differences were found in LVESV or LVEDV between the Sham and I/R + NV animals.

Histological analysis showed that infarct size (Fig. 7E and F) and the thickness of the infarcted wall (Fig. 7E and G) were significantly improved in the I/R + NV group compared to the I/R group at week 4. However, the infarct wall thickness remained significantly thinner in both groups that underwent I/R induction compared to the corresponding regions of the hearts from the Sham group. Additionally, treatment with ^B2MKO^hiPSC-NVs significantly reduced the proportion of TUNEL-positive CMs in mouse hearts on day 3 following I/R injury (Supplemental Fig. 18). Collectively, these observations indicated that treatment with ^B2MKO^hiPSC-NVs significantly improved cardiac function and reduced infarct size in the mouse hearts after I/R induction.

### 3.8 Internalization of ^B2MKO^hiPSC-NVs by recipient mouse CMs and induction of proliferation

**Fig. 8:**
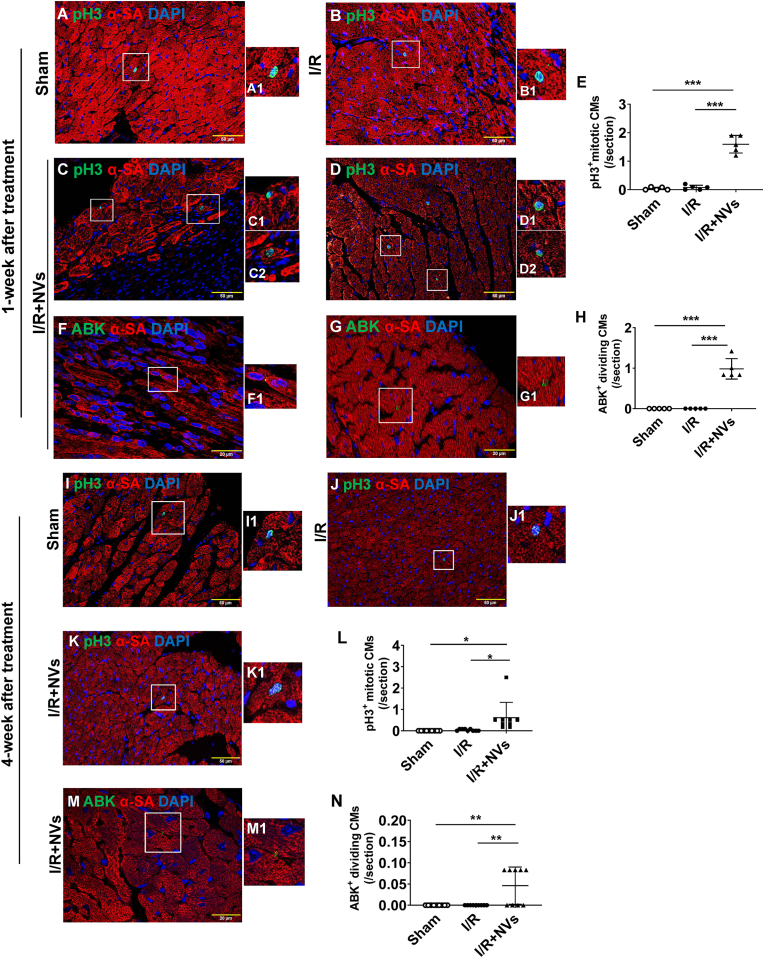
**^B2MKO^hiPSC-NV administration induces recipient CM proliferation after I/R induction in mouse hearts.** Representative images of pH3 immunofluorescence were displayed for sections from the corresponding region of the Sham hearts **(A)**, infarct border zone (BZ) of the I/R hearts **(B)**, infarct zone (IZ) **(C)** and BZ **(D)** of the I/R + NVs hearts. **(E)** The density of pH3^+^ CMs was calculated for each animal group**.** Representative images of ABK immunofluorescence were shown for sections from the IZ **(F)** and BZ **(G)** of the I/R + NVs mice hearts. **(H)** The density of CMs positive for ABK was calculated for each animal group. Representative images of pH3 immunofluorescence were displayed for sections from the corresponding region of the Sham hearts **(I)**, BZ of the I/R **(J)** and the I/R + NVs **(K)** hearts. **(L)** The density of pH3^+^ CMs was calculated for each animal group**.** Representative images of ABK immunofluorescence were displayed for sections from BZ **(M)** of the I/R + NVs mice hearts. **(N)** The density of ABK^+^ dividing CMs was calculated for each animal group. Boxed regions in panels A–D, F, G, I-K, and M were displayed at a higher magnification in A1-D1, F1, G1, I1-K1, and M1, respectively. N = 5 animals in each group for panels E and H; N = 9 animals in each group for panels L and N. Values were presented as mean ± SD. One-way ANOVA followed by the Tukey test: ∗*P* < 0.05, ∗∗*P* < 0.01, and ∗∗∗*P* < 0.001.

Injected ^B2MKO^hiPSC-NVs, labeled with PKH67, were not only found in the mouse myocardium but also internalized by the recipient mouse CMs (Supplemental Fig. 19) at Week 1 after I/R and NV injection. At week 1, recipient mouse CMs expressing pH3 or ABK were present in both the infarcted and the border zones of the I/R + NV-treated hearts (Fig. 8A–H and Supplemental Fig. 20). At week 4, these markers were detected in the border-zone sections of the I/R + NV-treated hearts (Fig. 8I–N and Supplemental Fig. 21). In contrast, both markers were nearly undetectable in CMs from the Sham or I/R mouse hearts, regardless of the zone or location. Consequently, the density of CMs exhibiting cell cycle activity was significantly greater in the I/R + NV-treated hearts compared to Sham or I/R hearts. These findings collectively indicated that hiPSC-NVs were internalized by mouse CMs and induced cell cycle activity in recipient CMs.

## 4 Discussion

The cardiogenic potential of hiPSCs is well-recognized, but their direct clinical application is limited by their pluripotency and the risk of tumor formation due to their unlimited capacity for self-renewal. As a result, hiPSCs have traditionally been used to generate CMs, smooth muscle cells, and endothelial cells for cardiac regenerative therapies. However, the clinical translation of hiPSC-derived cell therapy has been constrained by poor rates of cell engraftment. Thus, acellular products that replicate the regenerative potential of hiPSCs may offer a more viable option for clinical application [44].

Due to the low engraftment of transplanted cells, much of the therapeutic benefit of cell therapy is likely attributed to paracrine factors or EVs secreted by the cells. EVs have a well-established role in tissue regeneration [[45], [46], [47]]. However, since EVs are naturally produced acellular product secreted into the culture medium of cells, the isolation of these vesicles can be both time- and labor-intensive. Additionally, the yield of EVs from this process is low, making it a challenge to produce large quantities for therapeutic applications. More recently, intracellular vesicles (IVs), a subtype of artificial cell-derived vesicles, have been isolated using physical measurements and have shown efficacy in skin tissue regeneration [48]. The yield of NVs was found to be 16 times greater than that of EVs isolated using the traditional ultracentrifuge method. In contrast, NVs can be produced using an extruder with hydrophilic, micro-sized pores, allowing for a production scale that exceeds the yield of EVs by 52-fold (Fig. 1D). Compared to EVs or IVs, which contain cell-type specific molecules [[45], [46], [47]], NVs are more comprehensive, containing nearly all the signaling molecules found in the parental cells. This is evident in our analysis, where ∼83 % of the 4923 proteins identified in ^B2MKO^hiPSCs were also found in ^B2MKO^hiPSC-NVs. Notably, many molecules that were more prevalent in ^B2MKO^hiPSC-NVs than in ^B2MKO^hiPSCs, including ROS scavengers, cell-cycle regulators, spindle proteins, and chromosomal proteins. This underscores the potential of hiPSC-NVs for tissue protection and regeneration.

The H_2_O_2_ scavenging properties of ^B2MKO^hiPSC-NVs are largely attributed to the presence of NUDT2, PRDX2, PRDX6, and GSR proteins in the ^B2MKO^hiPSC-NVs. These proteins play a key role in directly scavenging H_2_O_2_, which helps to mitigate oxidative damage in hiPSC-CMs. Upon internalization by target cells, the proteins in ^B2MKO^hiPSC-NVs act in concert to enhance cell survival under hypoxic conditions and promote cellular proliferation, both in cultured hiPSC-CMs and in recipient CMs within infarcted mouse hearts. Both *in vitro* and *in vivo* experiments demonstrated that ^B2MKO^hiPSC-NVs can protect hiPSC-CMs from hypoxia-induced cell injury and apoptosis, as well as from I/R injury in mouse CMs. The protective effect of these NVs is significantly diminished when AKT activity is inhibited, further supporting the role of AKT signaling in mediating the cytoprotective effects of ^B2MKO^hiPSC-NV.

In addition to the 16 spindle proteins, 17 chromosomal proteins, and 24 cell-cycle proteins that were significantly enriched in ^B2MKO^hiPSC-NVs and may directly or indirectly contribute to CM proliferation, a synergistic effect from the proteins in the ^B2MKO^hiPSC-NV cargo not only upregulated YAP protein expression, but also facilitated the translocation of YAP into the nuclei of hiPSC-CMs. Inhibition of YAP function resulted in a significant reduction in YAP protein abundance in the nuclei of hiPSC-CMs, as well as a marked decreased in the proportion of hiPSC-CMs undergoing cytokinesis. Given that YAP is well-known to play a key role in cell proliferation [43], ^B2MKO^hiPSC-NVs likely target YAP signaling to promote CM proliferation.

The study by Lozano et al. characterized the proteomic profile of hiPSC-NVs and identified key proteins involved in various biological processes, including wound tissue repair (GJA1, HSP20/27/70, and HMGB1), wound healing (FLNA, MYH9, ACTC1, and ILK), stress response/translation initiation (eIF2S1/S2/S3/B4), hypoxia response (HMOX2, HSP90, and GNB1), and extracellular matrix organization (ITGA6, MFGE8, and ITGB1) [26]. *In vitro*, these NVs promoted angiogenesis in endothelial cells, enhanced the survival of CMs exposed to hypoxia, and reduced cardiac fibroblast activation. Our current study aligns with Lozano's findings, demonstrating that ^B2MKO^hiPSC-NVs similarly promoted CM survival under hypoxic conditions. In addition to exploring the proteomic profile of ^B2MKO^hiPSC-NVs, we also investigated the potential mechanisms and efficacy of ^B2MKO^hiPSC-NV administration for CM protection and cell cycle induction, both *in vitro* and in a mouse model of I/R *in vivo*. We identified four ROS scavengers, as well as 16 spindle proteins, 17 chromosomal proteins, and 24 cell-cycle proteins that were highly enriched in ^B2MKO^hiPSC-NVs, all of which may contribute to CM protection and the promotion of cell cycle activity.

The results from our *in vitro* experiments with CD8^+^ T cell and *in vivo* injections of hiPSC-NVs confirmed that B2MKO reduced treatment-related immunogenicity compared to ^WT^hiPSC-NVs. Additionally, ^B2MKO^hiPSC-NVs expressed CD47, which inhibits natural killer cells [42], further suggesting that both ^B2MKO^hiPSCs and ^B2MKO^hiPSC-NVs are expected to be less immunogenic during clinical use. Give the time required for hiPSC reprogramming and NV manufacturing, autologous hiPSC-NVs may not be practical for emergency situations, such as acute MI. However, ^B2MKO^hiPSC-NVs offer the advantage of hypoimmunogenic properties, allowing patients to receive allogeneic hiPSC-NVs that have been pre-manufactured. Furthermore, ^B2MKO^hiPSC-NVs maintain their cytoprotective properties even after prolonged cryopreservation, potentially enabling their use as a commercially viable product for long-term storage. This ^B2MKO^hiPSC line facilitates the large-scale production of hypoimmunogenic hiPSC-NVs, which could be suitable for allogeneic transplantation in patients with varying immune histocompatibility. These NVs could be used in clinical settings, such as open chest surgery or percutaneous transluminal coronary angioplasty, for cardiac protection and CM regeneration in patients with ischemic heart disease or MI.

In conclusion, NVs derived from ^B2MKO^hiPSCs exhibited hypoimmunogenic properties, promoted proliferation of both hiPSC-CMs and mouse CMs, and enhanced resistance to hypoxia- and ROS-induced damage. These NVs also led to significant improvements in functional recovery and reduction in infarct size in a mouse heart model of I/R injury. Collectively, these findings support the potential of this emerging strategy for CM protection and regeneration in the treatment of MI.

## CRediT authorship contribution statement

**Yuhua Wei:** Writing – review & editing, Methodology, Investigation, Formal analysis, Data curation. **Xiaoxiao Geng:** Investigation, Methodology, Data curation. **Qing You:** Conceptualization, Investigation, Methodology. **Yu Zhang:** Investigation, Formal analysis, Data curation. **Fangfang Cao:** Methodology. **Gunaseelan Narayanan:** Methodology. **Thanh Nguyen:** Investigation, Methodology. **Xiaoyuan Chen:** Methodology, Writing – review & editing. **Jianyi Zhang:** Writing – review & editing, Resources, Project administration, Funding acquisition. **Lei Ye:** Conceptualization, Investigation, Methodology, Formal analysis, Data curation, Project administration, Writing – original draft, Writing – review & editing.

## Data availability

The data supporting the findings of this article are available within the article itself and its supplementary online material.

## Ethics approval and consent to participate

All animal procedures and protocols involved were approved by the Institutional Animal Care and Use Committee of the University of Alabama at Birmingham, USA and performed according to the guidelines of the National Institutes of Health (NIH publication No 85-23).

## Funding

This study was supported in part by the 10.13039/100000050National Heart, Lung, and Blood Institute Grant Numbers: U01HL134764, 10.13039/100000002NIH P01 HL160476 and R01HL131017, R01HL149137.

## Declaration of competing interest

X.C. is a co-founder of and holds shares in Yantai Lannacheng Biotechnology Co., Ltd. Other authors have nothing to declare.
