# Supplementary material for: Human induced pluripotent stem cell derived nanovesicles for cardiomyocyte protection and proliferation
Source: Bioact Mater. 2025 May 2;50:585–602. doi: 10.1016/j.bioactmat.2025.04.017 (PMC12124652; doi:10.1016/j.bioactmat.2025.04.017)
Supplement: Multimedia component 1 [file mmc1.pdf]

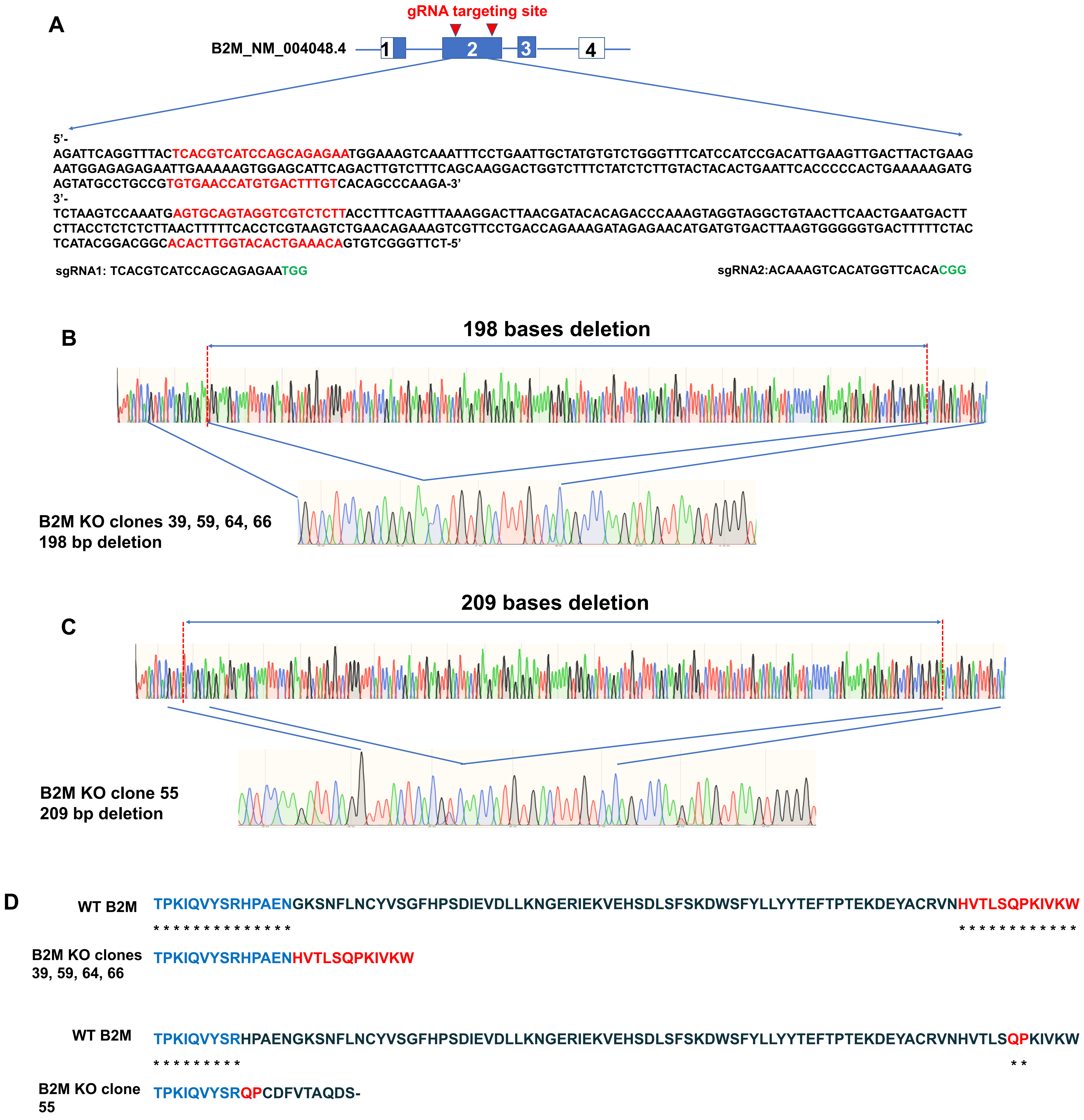

Supplemental Figure 1. B2MKO mutation was induced *via* deletion of 198- or 209-bp in exon 2 of B2M. (A) The CRISPR/Cas9 deletion strategy is displayed as a schematic illustration; guide RNA (gRNA) binding sites are identified with red arrowheads and the corresponding sequences are displayed in red font. **(B)** Sequencing chromatograms are displayed for the wild-type (WT) B2M sequence and the sequence in clones 39, 59, 64, and 66 confirmed that the 198-bp deletion was successful. **(C)** Sequencing chromatograms are displayed for the WT B2M sequence and the sequence in clone 55 confirmed that the 209-bp deletion was successful. **(D)** The B2M protein sequences in clones 39, 59, 64, 66, and 55 were aligned with the WT B2M protein sequence.

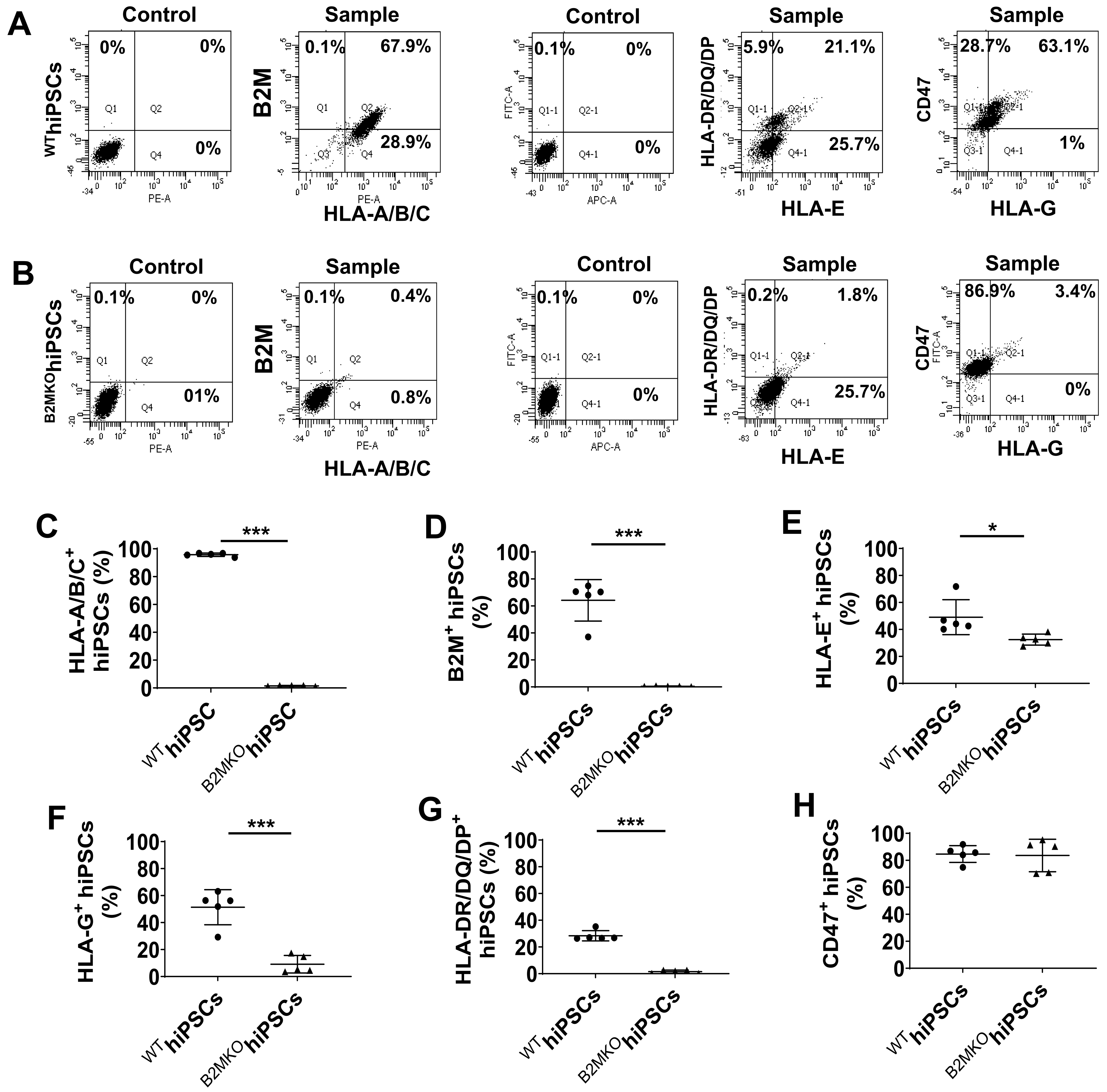

Supplemental Figure 2. Expression of histocompatibility proteins was significantly reduced in ^{B2MKO}hiPSCs. Protein expressions of HLA-A/B/C, B2M, HLA-E, HLA-G, HLA-DR/DQ/DP, and CD47 in **(A)** ^{WT}hiPSCs and **(B)** ^{B2MKO}hiPSCs were evaluated by flow cytometry. The proportion of cells expressing **(C)** HLA-A/B/C, **(D)** B2M, **(E)** HLA-E, **(F)** HLA-G, **(G)** HLA-DR/DQ/DP, and **(H)** CD47 was quantified and presented as a percentage. Data were presented as mean ± SD. N = 5 biological replicates. Unpaired T-Test, * P<0.05 and ****P* < 0.001.

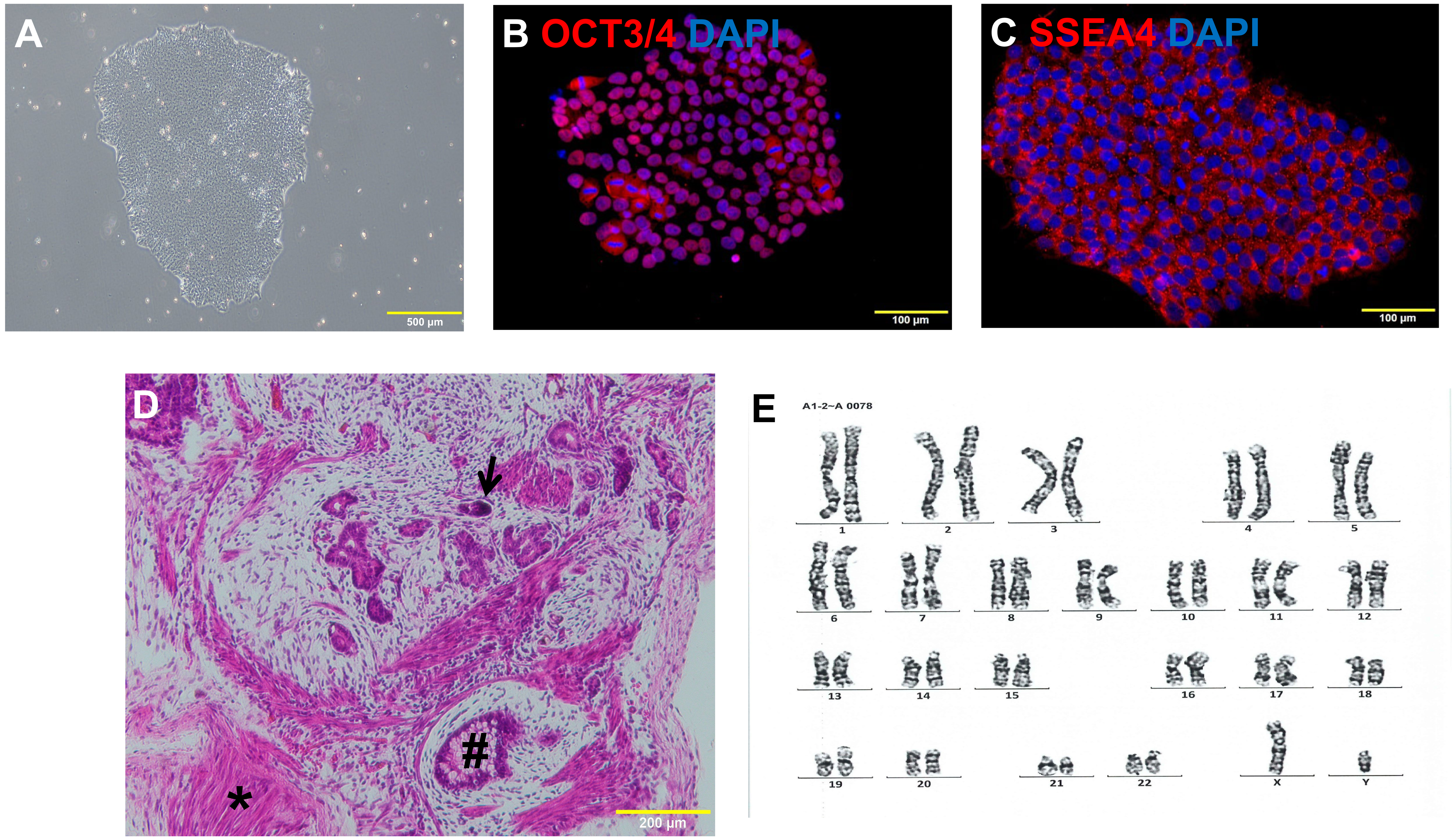

Supplemental Figure 3. ^{B2MKO}hiPSCs were morphologically normal and pluripotent with a normal karyotype. (A) A phase-contrast imaging and **(B-C)** immunofluorescence imaging of **(B)** OCT3/4 and **(C)** SSEA4 protein expression were performed with ^{B2MKO}hiPSCs colonies. Nuclei were counterstained with DAPI in panels **B** and **C**. **(D)** Two months after ^{B2MKO}hiPSCs were injected into NOD-SCID immunodeficient mice, a teratoma was explanted, paraffin-embedded, sectioned, and stained with hematoxylin and eosin to identify muscle (*, mesoderm), epidermal tissue (↑, ectoderm), and gastrointestinal glands (#, endoderm). **(E)** The Chromosomal structure of ^{B2MKO}hiPSCs during metaphase was evaluated *via* karyotype analysis.

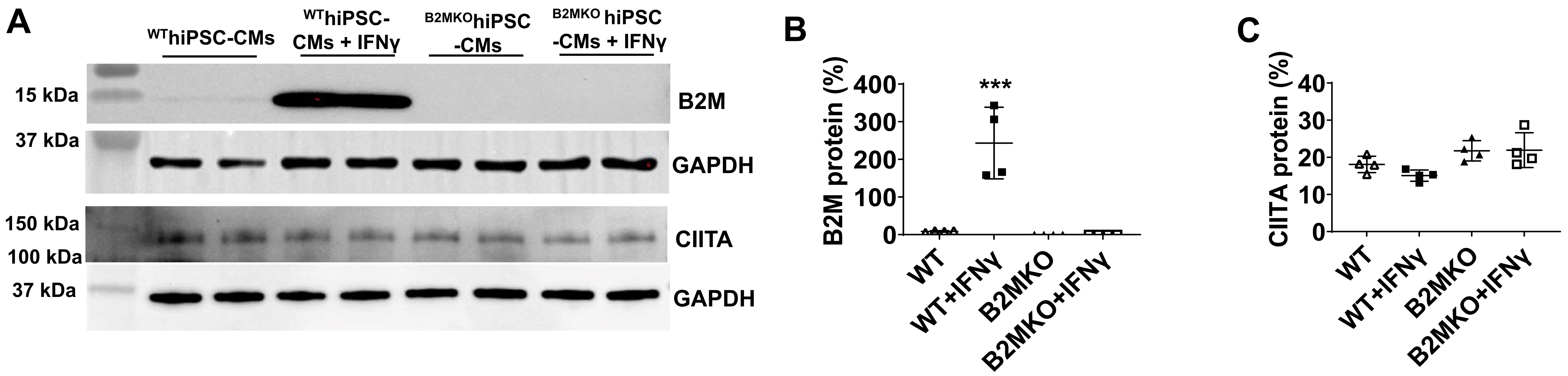

Supplemental Figure 4. β2-microglobin (B2M) knockout (B2MKO) suppressed the immune response to interferon γ treatment in hiPSC-CMs. WThiPSCs and B2MKOhiPSCs were differentiated into CMs and cultured with or without 25 ng/mL interferon γ (IFNγ) for 48 hours. **(A)** Protein expression levels of B2M and Class II transactivator (CIITA) were evaluated by Western Blot. Quantification of **(B)** B2M and **(C)** CIITA protein levels *via* Western Blot. Each experiment conducted with 4 biological replicates. Data were presented as mean ± SD. One-way ANOVA followed by Tukey's test: ***P < 0.001 vs all other sample.

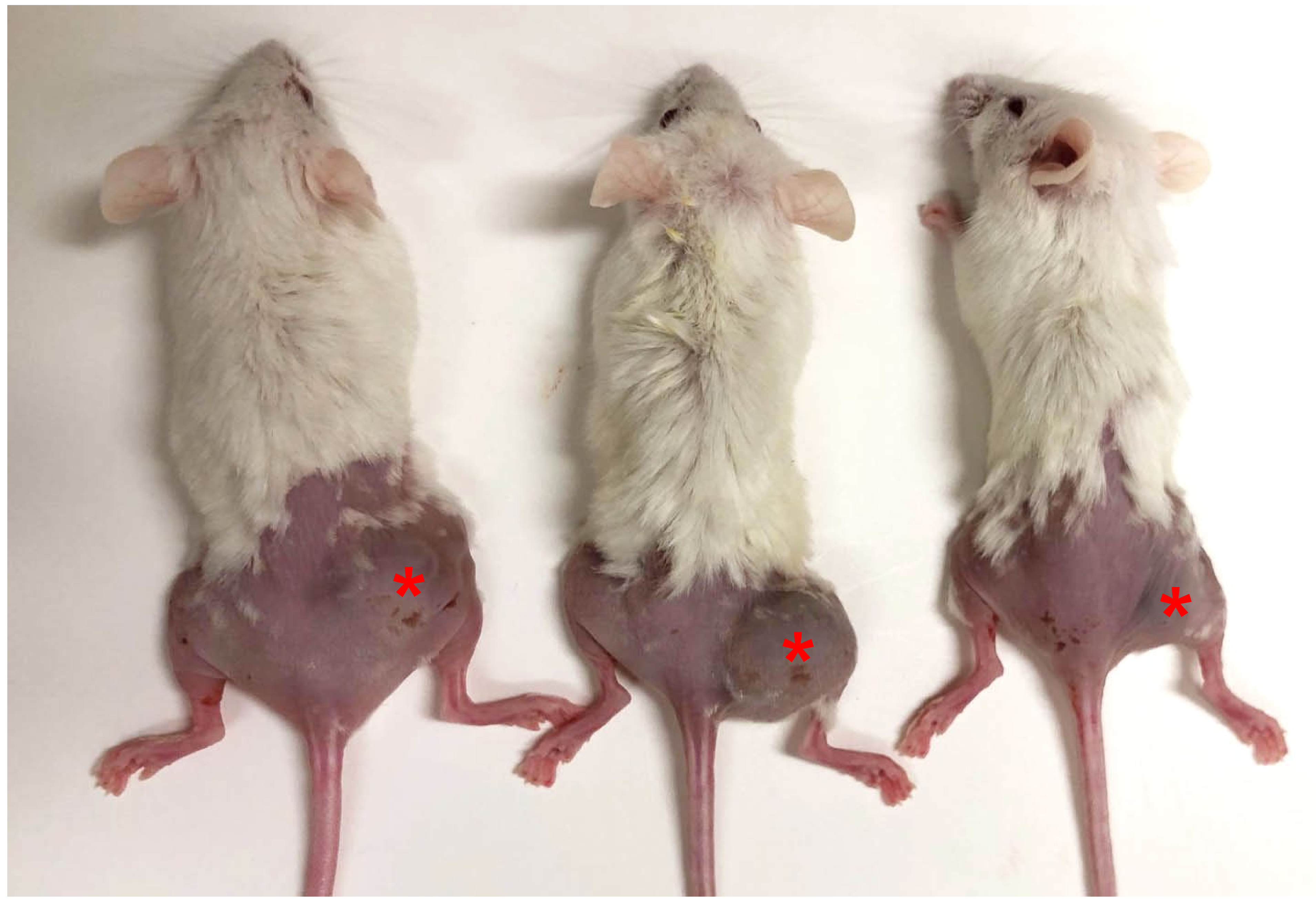

Supplemental Figure 5. ^{B2MKO}hiPSC-NVs did not induce teratoma formation. NOD-SCID received subcutaneous injections of 3 x 10^{11} ^{B2MKO}hiPSC-NVs into the left flank and 2 x 10^{6} ^{B2MKO}hiPSCs into the right flank. One and a half months later, a teratoma (*) formed in the right flank, while no tumor was detected in the left flank.

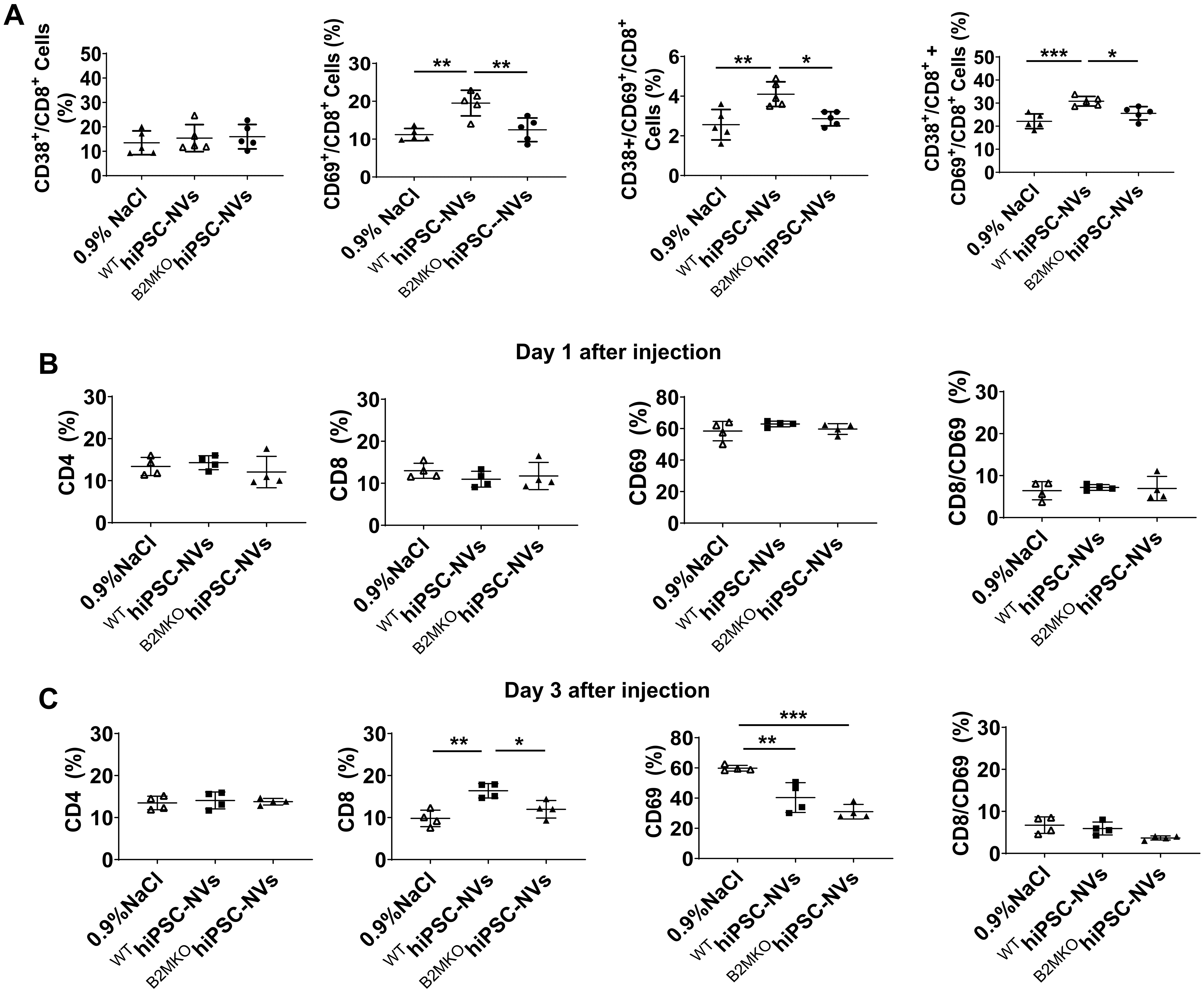

Supplemental Figure 6. B2MKOhiPSC-NVs were less immunogenic than WThiPSC-NVs. **(A)** *In vitro*, the proportions of CD8+ T cells that expressed either CD38, CD69 or co-expressed both CD38 and CD69 were determined by flow cytometry and expressed as a percentage (n = 5 biological replicates). **(B & C)** *In vivo*, the proportions of T lymphocytes expressing CD4, CD8, CD69, or co-expressing both CD8 and CD69 on days 1 **(B)** and 3 **(C)** after i.p. injection in C57BL mice were determined *via* flow cytometry and expressed as a percentage (n = 4 animals per group). Data were presented as mean ± SD. One-way ANOVA followed by the Tukey test. $P < 0.05$, $^{**}P < 0.01$, and $^{***}P < 0.001$.

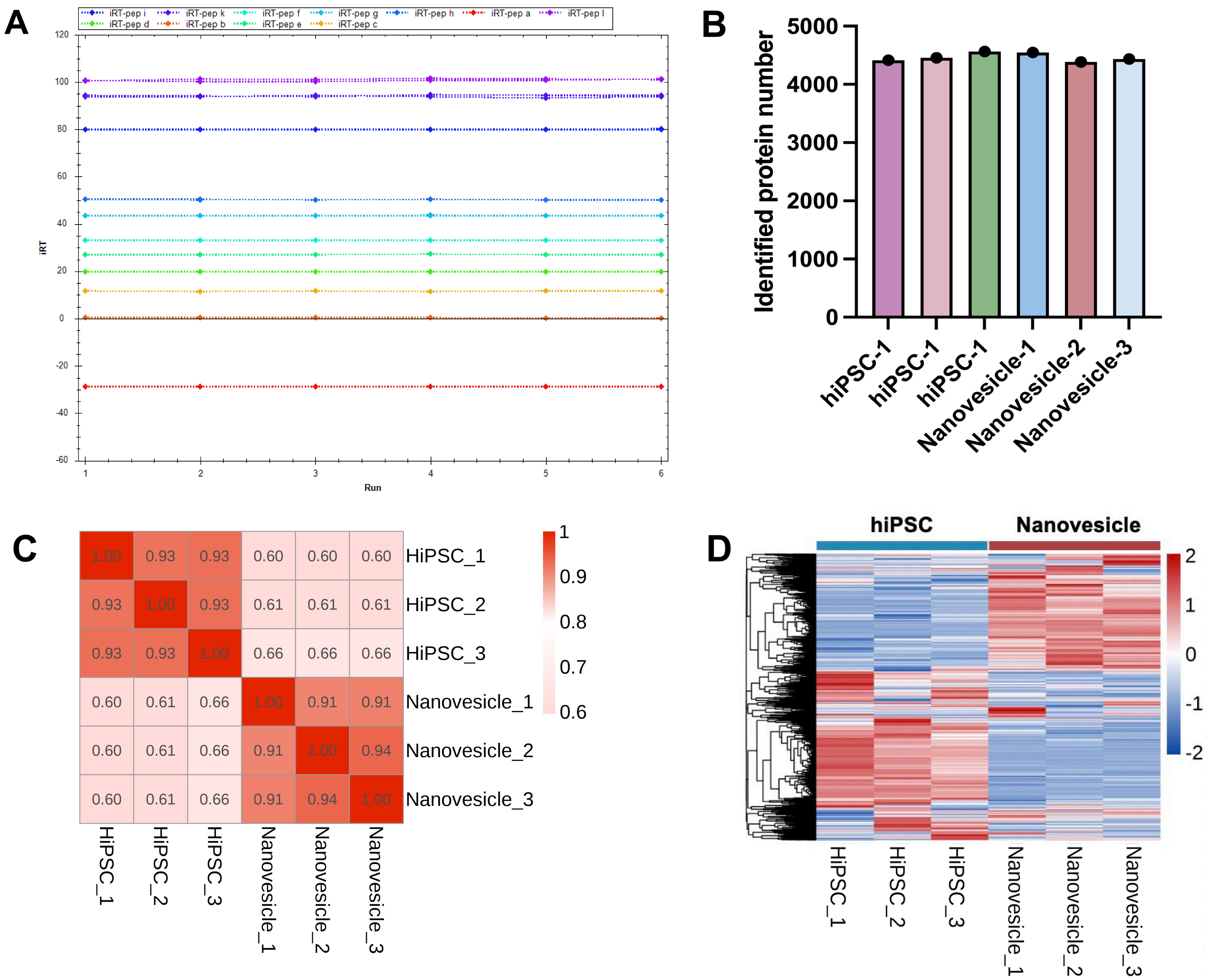

Supplemental Figure 7. Proteomic atlas of B2MKOhiPSCs and B2MKOhiPSC-NVs. **(A)** Indexed retention time values of the internal standard calibration peptide. **(B)** Histogram showing the count of identified proteins in B2MKOhiPSCs and B2MKOhiPSC-NVs. **(C)** Spearman correlation of the identified proteins in B2MKOhiPSCs and B2MKOhiPSC-NVs were calculated to assess the robustness of label-free quantification. **(D)** Heatmap of identified proteins in B2MKOhiPSCs and B2MKOhiPSC-NVs. (n = 3 biological replicates).

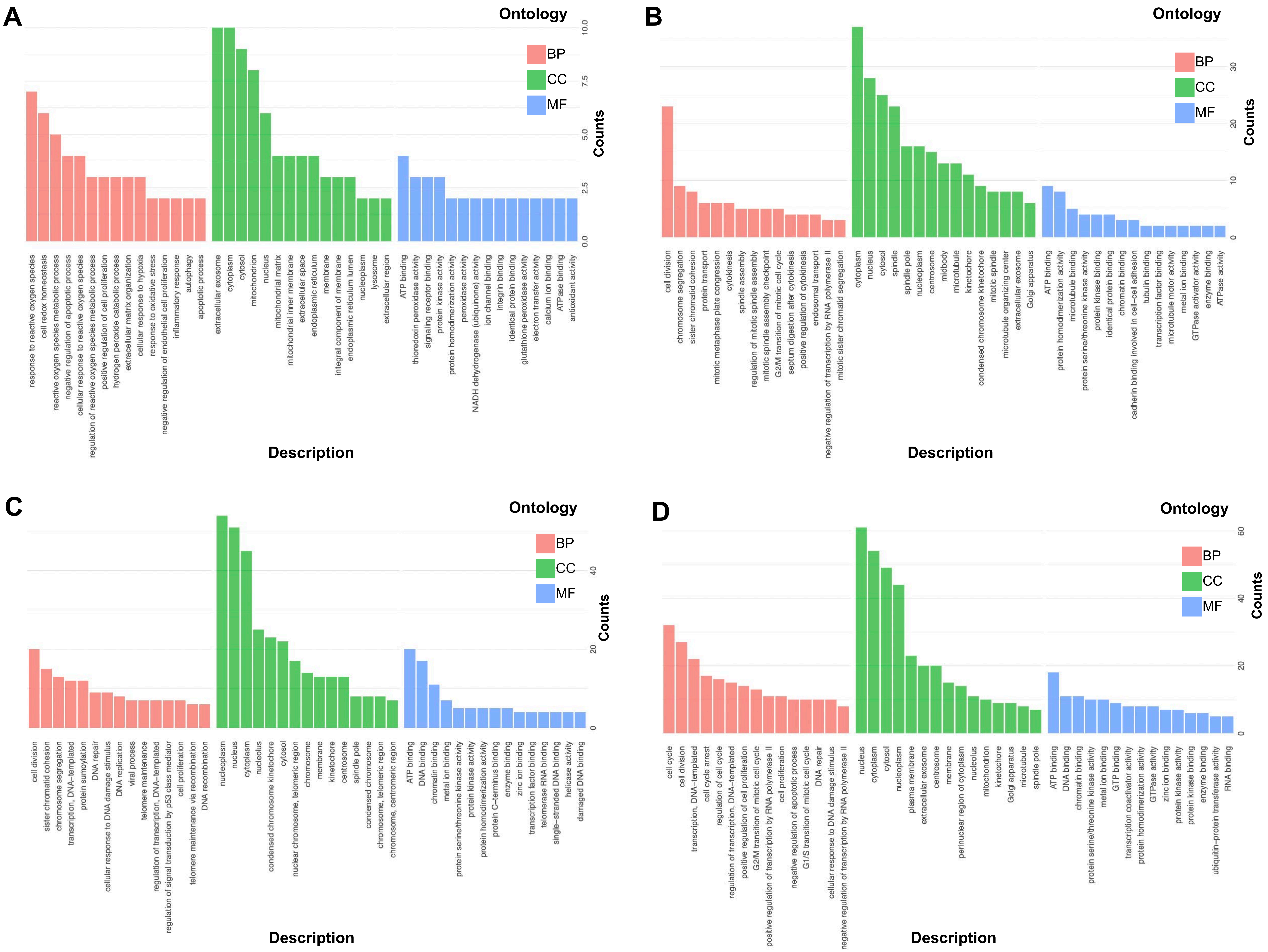

Supplemental Figure 8. Gene Ontology (GO) enrichment analysis. The figure represented the enrichment analysis of biological process (BP), cellular component (CC), and molecular function (MF) for co-expressed proteins related to ROS **(A)**, spindle **(B)**, chromosome **(C)** and the cell cycle **(D)** in ^{B2MKO}hiPSCs and ^{B2MKO}hiPSC-NVs. (n = 3 biological replicates).

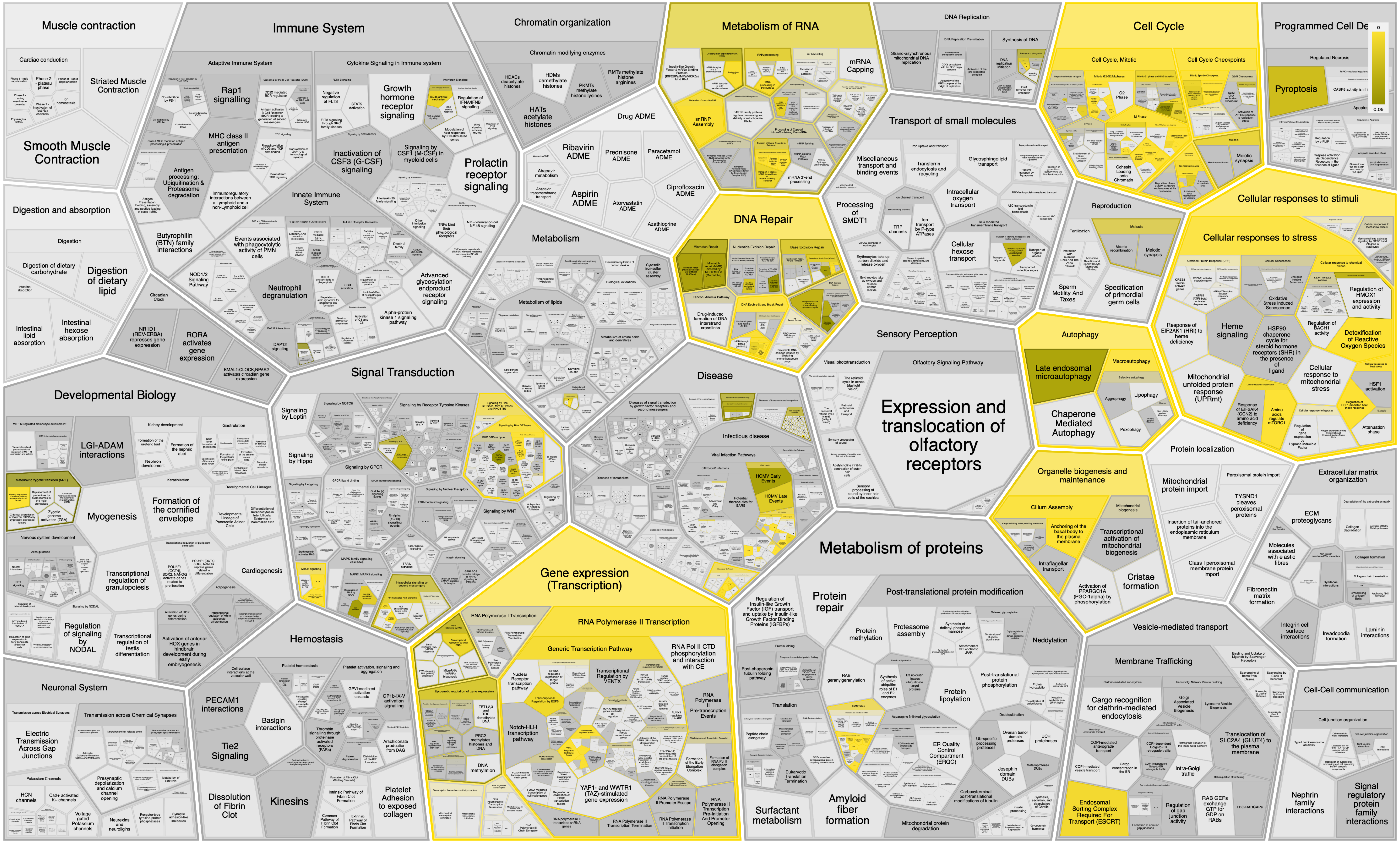

Supplemental Figure 9. Voronoi diagram overview of signaling pathways based on Reactome analysis of ROS, spindle, chromosome, and the cell cycle-related proteins in ^{B2MKO}hiPSCs and ^{B2MKO}hiPSC-NVs.

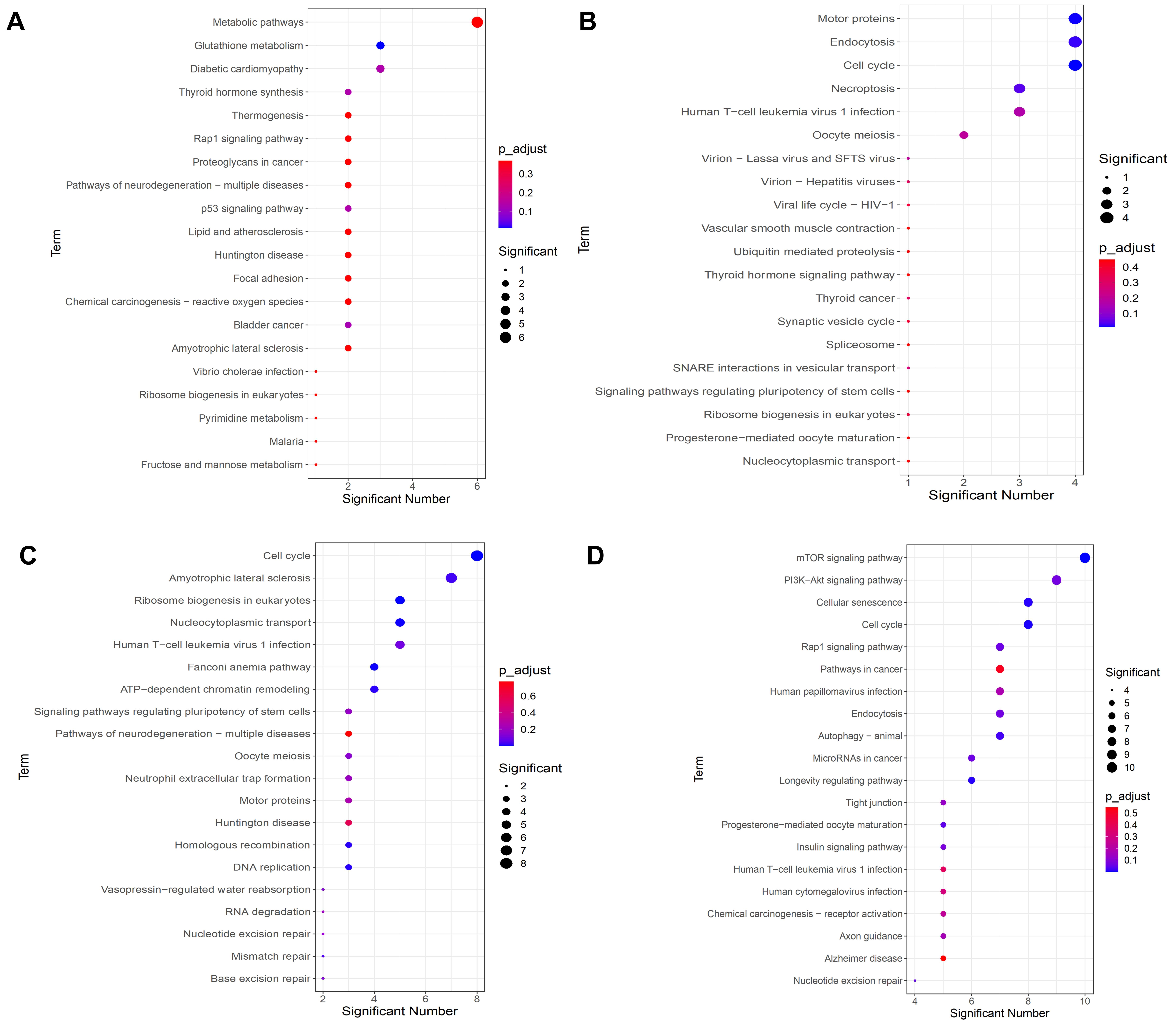

Supplemental Figure 10. Kyoto Encyclopedia of Gene and Genomes (KEGG) enrichment analysis. KEGG pathways for co-expressed proteins related to ROS **(A)**, spindle **(B)**, chromosome **(C),** and the cell cycle **(D)** in ^{B2MKO}hiPSCs and ^{B2MKO}hiPSC-NVs. (n = 3 biological replicates).

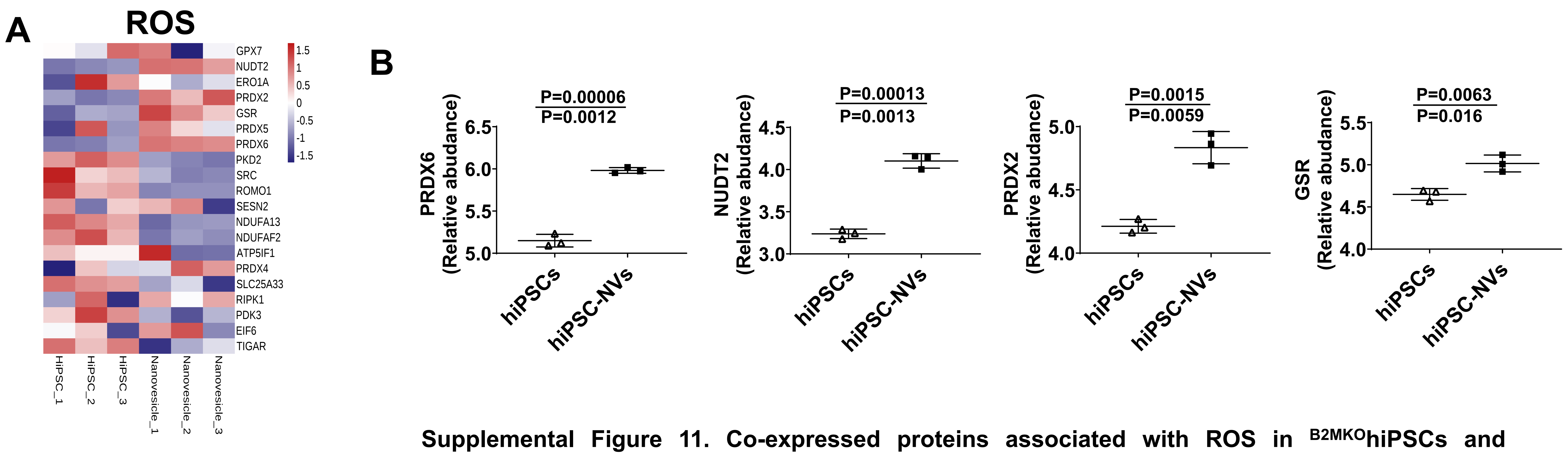

Supplemental Figure 11. Co-expressed proteins associated with ROS in B2MKOhiPSCs and B2MKOhiPSC-NVs. (A) Heatmap of co-expressed proteins associated with ROS in B2MKOhiPSCs and B2MKOhiPSC-NVs. **(B)** Top 4 anti-ROS-related proteins were more abundant in B2MKOhiPSC-NVs than in B2MKOhiPSCs. Raw protein abundance data were log10-transformed and analyzed using a two-tailed unpaired T-Test followed by Benjamini-Hochberg correction. P values above the line corresponded to the two-tailed unpaired T-Test results, while P values below the line represented the results after Benjamini-Hochberg correction. (n = 3 biological replicates).

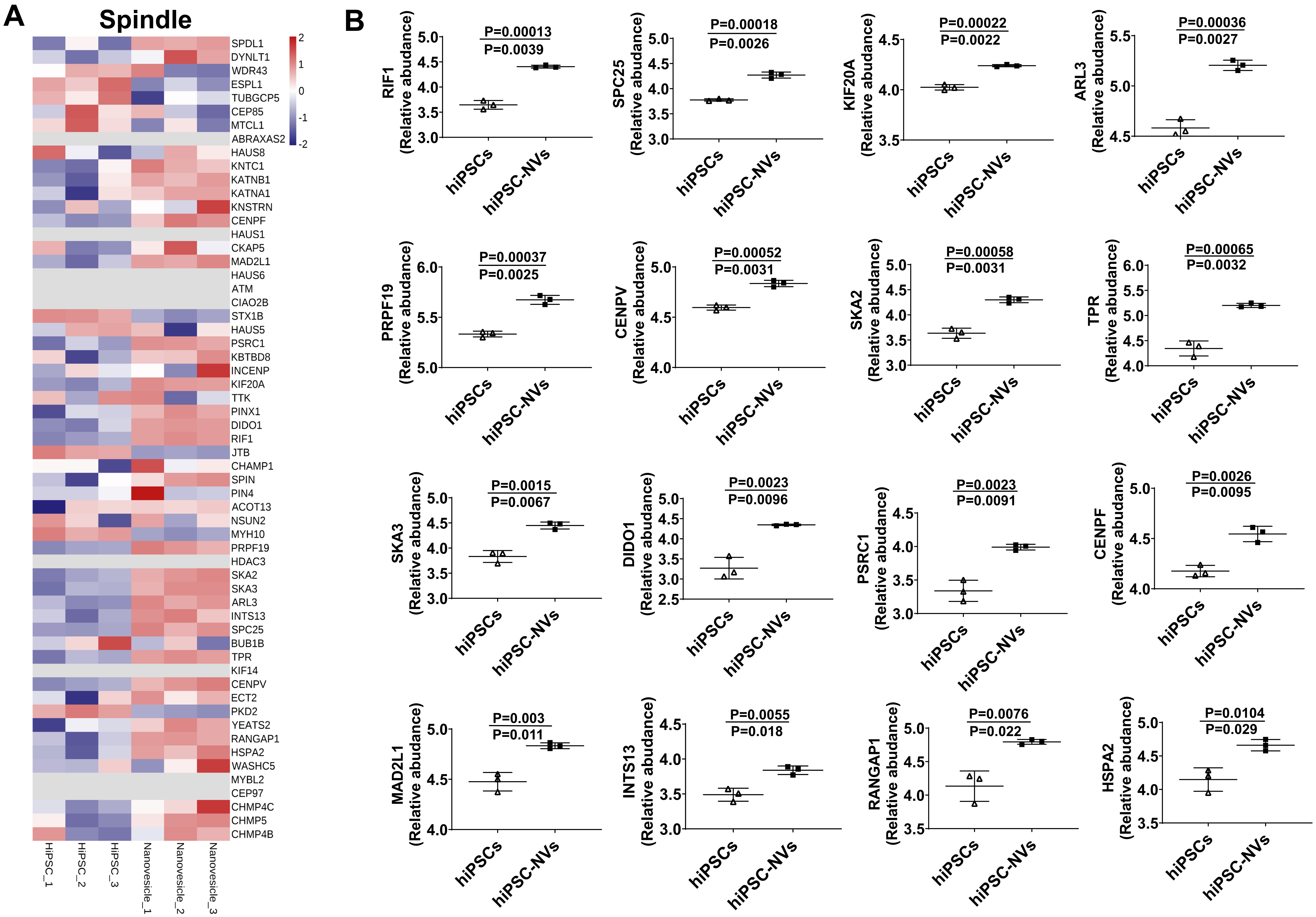

Supplemental Figure 12. Co-expressed proteins associated with the spindle in ^{B2MKO}hiPSCs and ^{B2MKO}hiPSC-NVs. (A) Heatmap of co-expressed proteins associated with the spindle in ^{B2MKO}hiPSCs and ^{B2MKO}hiPSC-NVs. **(B)** Top 16 spindle-related proteins that were more abundant in ^{B2MKO}hiPSC-NVs than in ^{B2MKO}hiPSCs. Raw protein abundance data were log10-transformed and analyzed using a two-tailed unpaired T-Test followed by Benjamini-Hochberg correction. P values above the line corresponded to the two-tailed unpaired T-Test results, while P values below the line represented results after Benjamini-Hochberg correction. (n = 3 biological replicates).

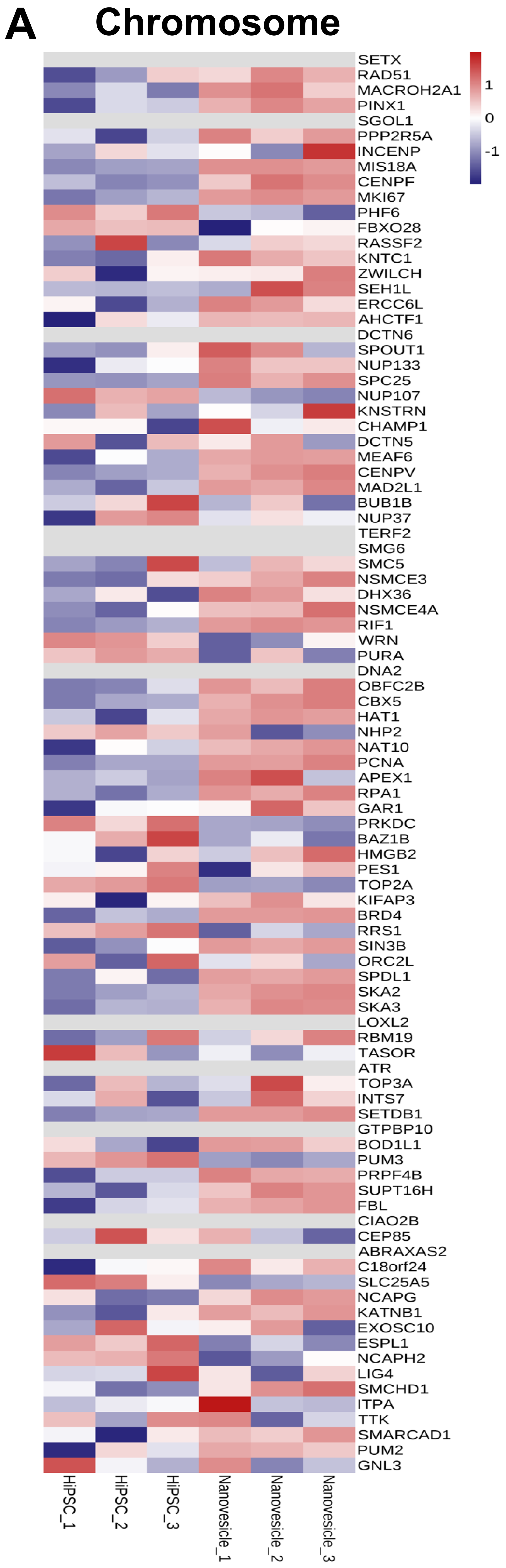

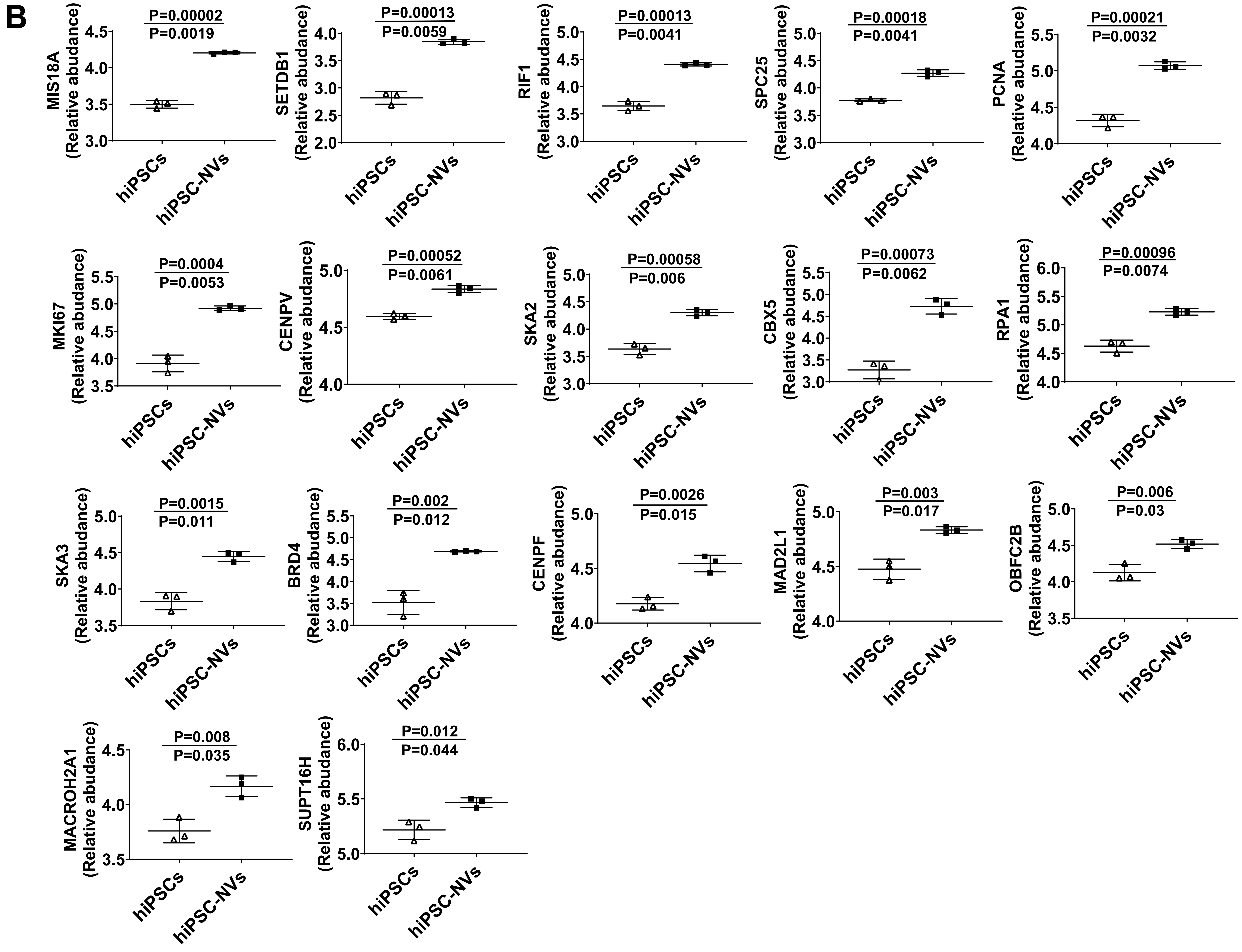

Supplemental Figure 13. Co-expressed proteins associated with chromosomes in B2MKOhiPSCs and B2MKOhiPSC-NVs. (A) Heatmap of co-expressed proteins associated with chromosomes in B2MKOhiPSCs and B2MKOhiPSC-NVs. **(B)** Top 17 chromosome-related proteins that were more abundant in B2MKOhiPSC-NVs than in B2MKOhiPSCs. Raw protein abundance data were log10-transformed and analyzed using a two-tailed unpaired T-Test followed by Benjamini-Hochberg correction. P values above the line corresponded to the two-tailed unpaired T-Test results, while P values below the line represented results after Benjamini-Hochberg correction. (n = 3 biological replicates).

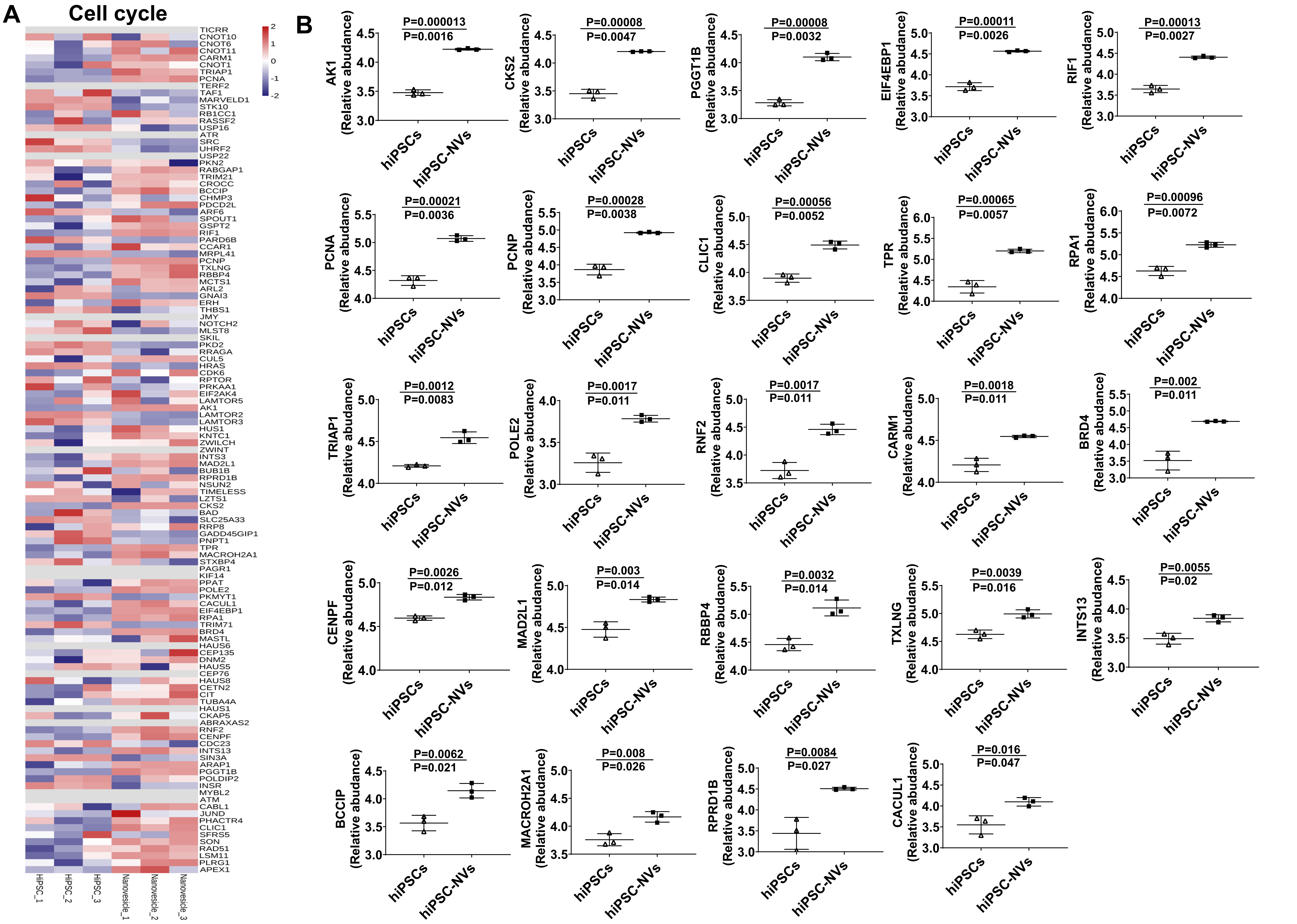

Supplemental Figure 14. Co-expressed proteins associated with the cell cycle in B2MKOhiPSCs and B2MKOhiPSC-NVs. (A) Heatmap of co-expressed proteins associated with the cell cycle in B2MKOhiPSCs and B2MKOhiPSC-NVs. **(B)** Top 24 cell cycle-related proteins that were more abundant in B2MKOhiPSC-NVs than in B2MKOhiPSCs. Raw protein abundance data were log10-transformed and analyzed using a two-tailed unpaired T-Test followed by Benjamini-Hochberg correction. P values above the line corresponded to the two-tailed unpaired T-Test results, while P values below the line represented results after Benjamini-Hochberg correction. (n = 3 biological replicates).

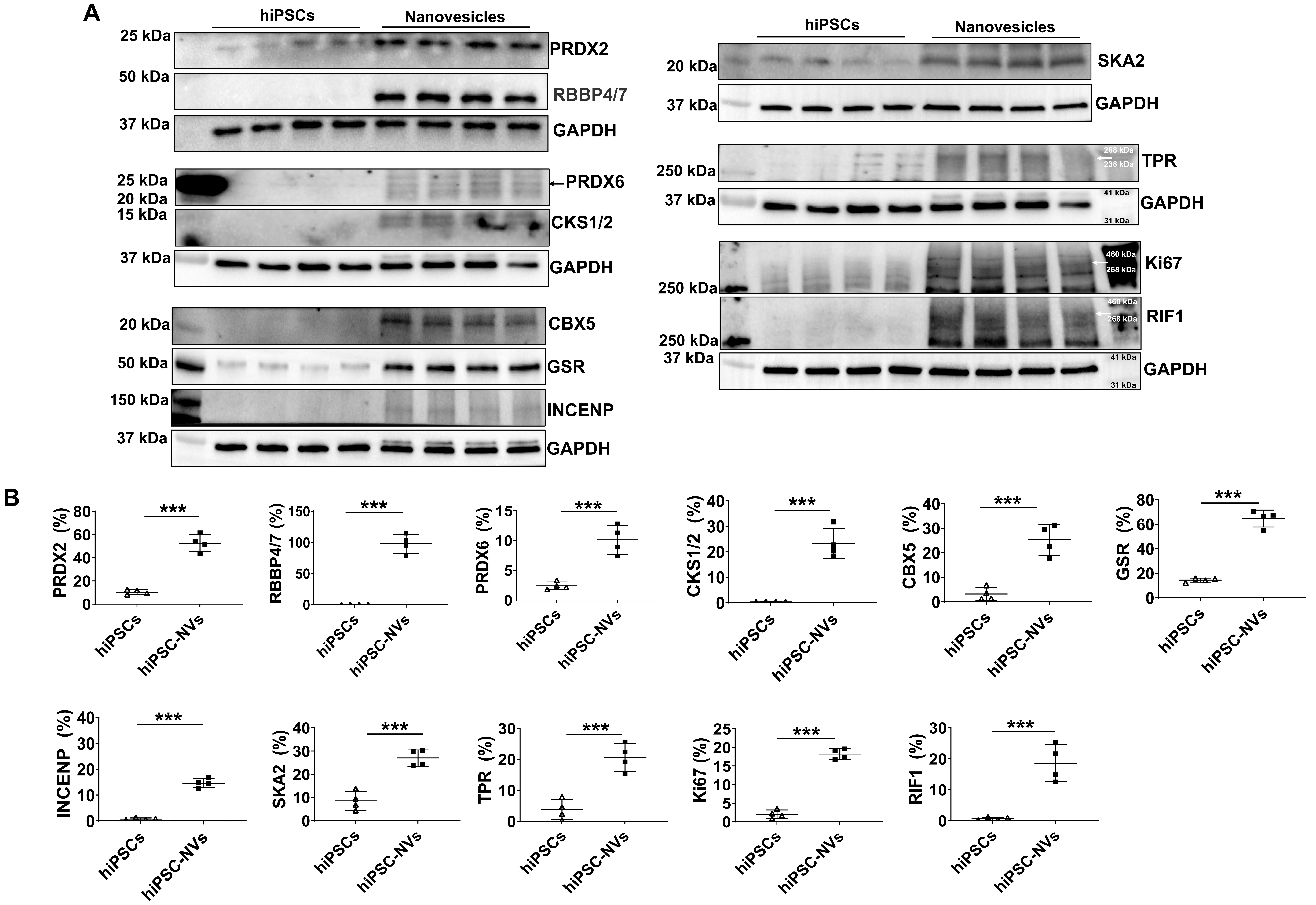

Supplemental Figure 15. Validation of proteins which are more abundant in B2MKOhiPSCs derived nanovesicles (hiPSC-NVs) than in B2MKOhiPSCs. (A) Representative Western Blot images of proteins involved in biological processes related to anti-ROS (PRDX2, PRDX6, and GSR), spindle (TPR, SKA2, RIF1, INCENP), chromosome (SKA2, RIF1, Ki67, CBX5, and INCENP), and the cell cycle (RBBP4/7, CKS1/2, RIF1, and TPR). **(B)** Quantification of validated proteins, which were expressed as percentages after normalization to GAPDH protein levels. (n = 4 biological replicates). Two-tailed unpaired T-Test: *** *p*-value < 0.001. Arrows indicated the respective protein bands.

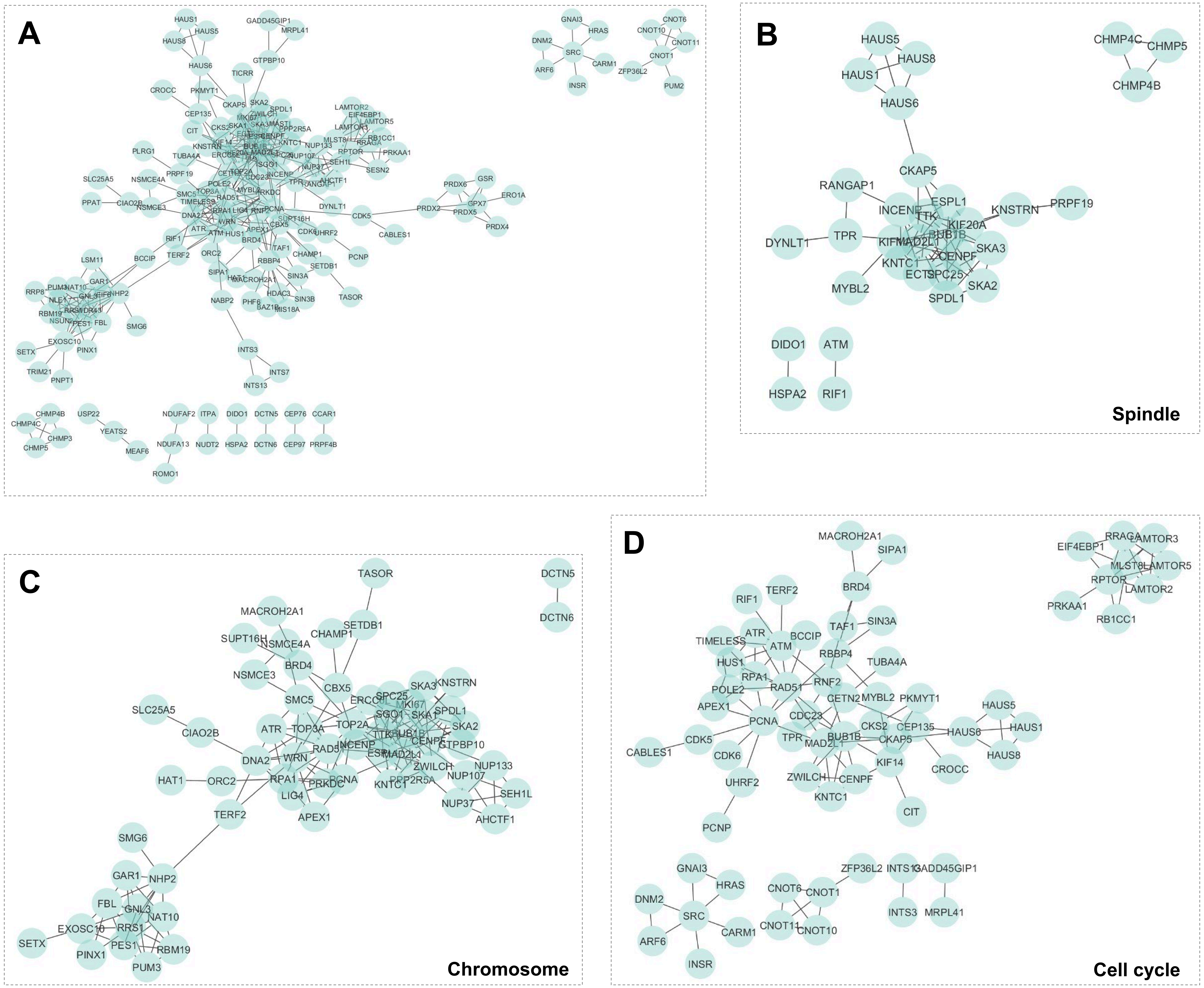

Supplemental Figure 16. Protein-protein interaction (PPI) network. **(A)** PPI network of ROS, spindle, chromosome, and the cell cycle-related proteins. PPI network of proteins involved in spindle **(B)**, chromosome **(C)** and the cell cycle **(D)** biological processes.

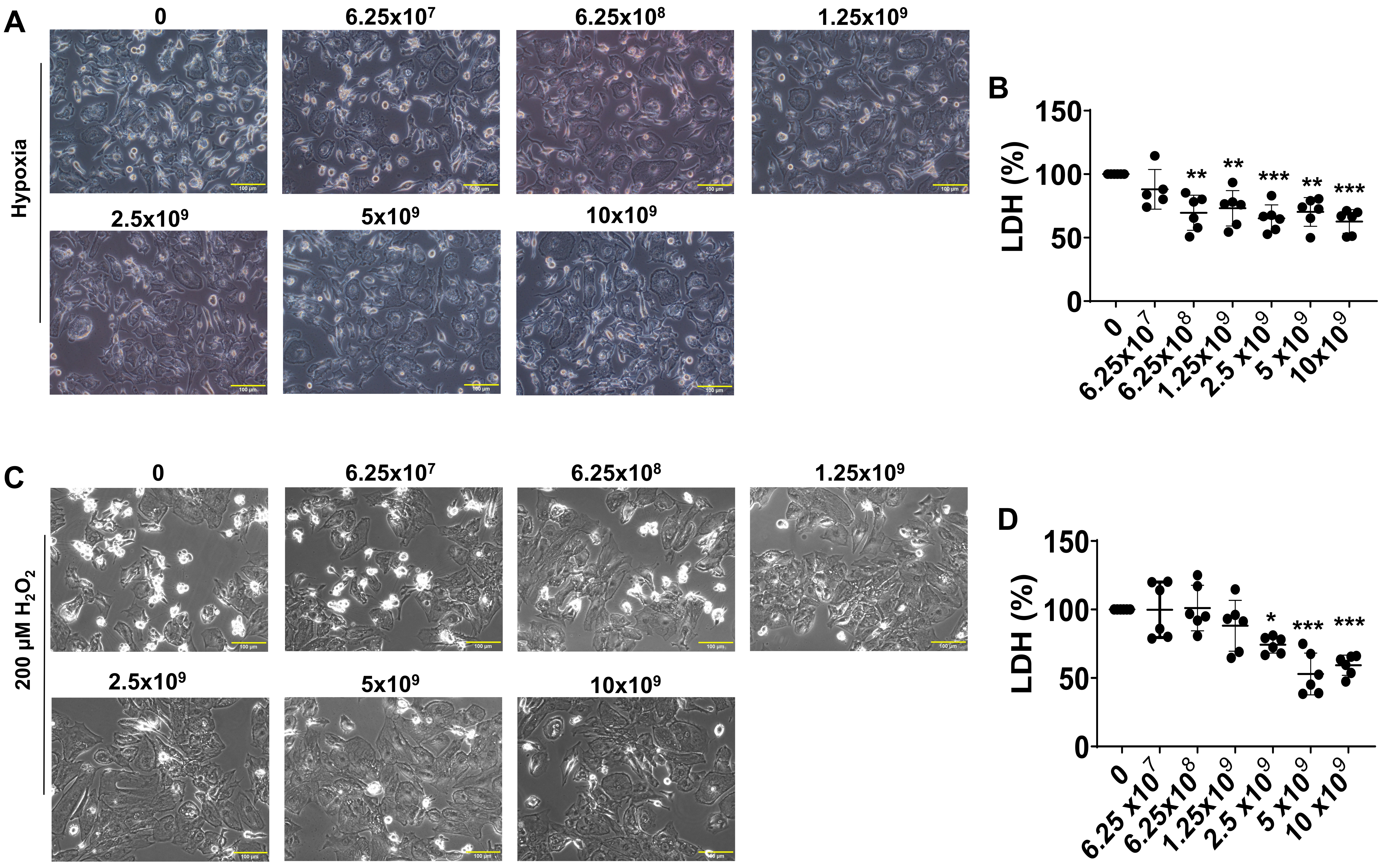

Supplemental Figure 17. Cryo-preserved ^{B2MKO}hiPSC-NVs, stored at -80°C for 3 weeks, improved ^{B2MKO}hiPSC-CM viability under hypoxic conditions and in the presence of H_2O_2. (A) ^{B2MKO}hiPSC-CMs were cultured under hypoxic conditions in the HBSS supplemented with the indicated concentrations of ^{B2MKO}hiPSC-NVs, which were stored at -80°C for 3 weeks, for 24 hours. **(B)** LDH levels in the cultured medium were measured, normalized to the levels in the absence of ^{B2MKO}hiPSC-NVs, and presented as a percentage (n=5-6 biological replicates). **(C)** ^{B2MKO}hiPSC-CMs were cultured in 200 µM H_2O_2, which was premixed with the indicated concentrations of ^{B2MKO}hiPSC-NVs, for 30 min. **(D)** Medium LDH levels were measured, normalized to the levels in the medium with 200 µM H_2O_2 in the absence of ^{B2MKO}hiPSC-NVs, and presented as a percentage (n=6 biological replicates). Panels A and C displayed representative images from each experiment, and values were presented as mean ± SD. Statistical analysis in panels B and D was performed using one-way ANOVA followed by the Tukey's test: *P < 0.05, **P < 0.01, and ***P < 0.001 vs 0 NV.

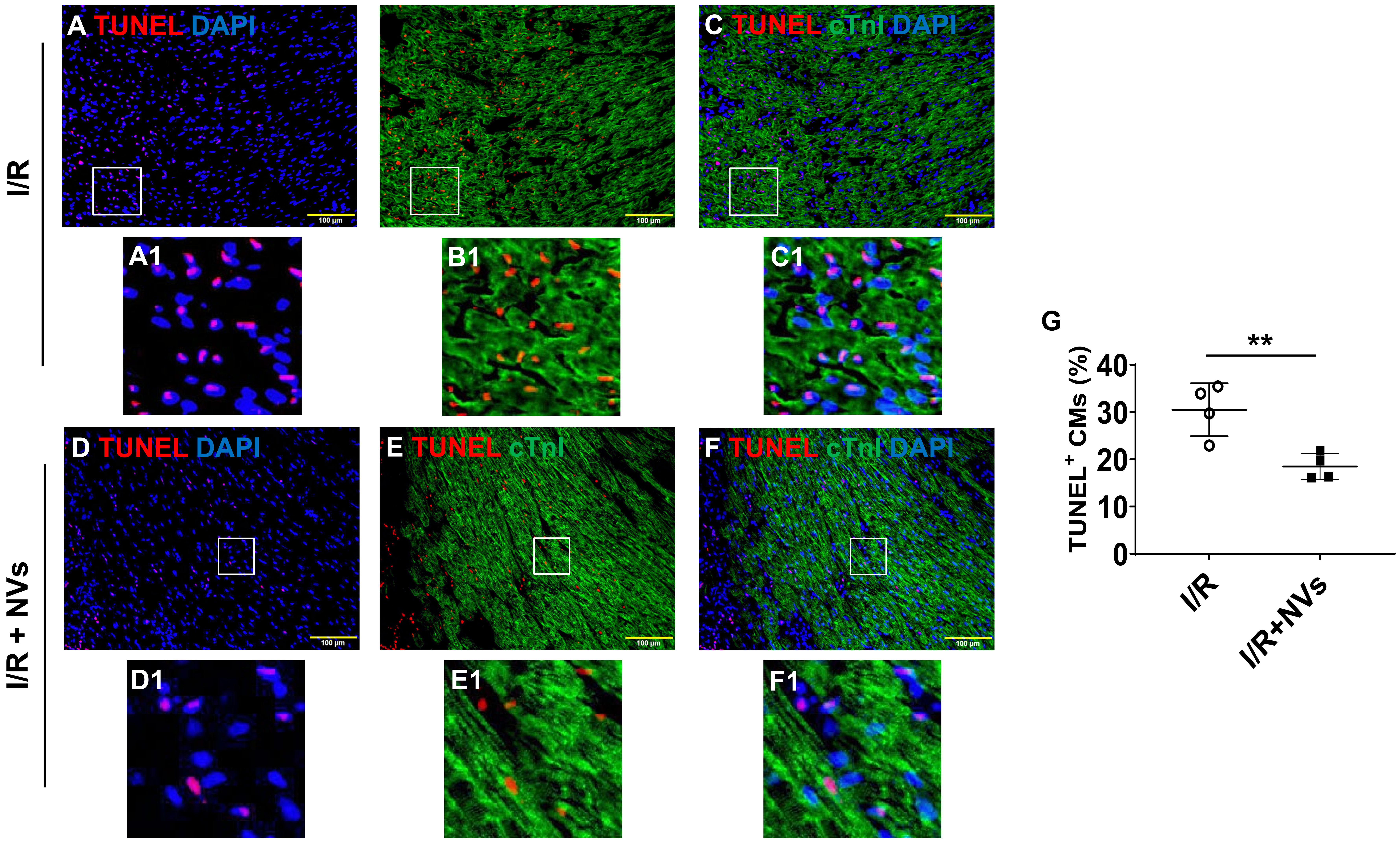

Supplemental Figure 18. B2MKOhiPSC-NV administration reduced CM apoptosis in mouse hearts with I/R injury. (A-C) Cardiac tissues were collected from the BZ region of the hearts from animals in the I/R group that were sacrificed 3 days after I/R induction. **(A)** TUNEL staining was performed, and nuclei were counterstained with DAPI. **(B)** CMs were visualized by staining for cardiac troponin I (cTnI). **(C) T**he images were merged to identify TUNEL-positive CMs. **(D-F)** Cardiac tissues were collected from the BZ region of the hearts from animals in the I/R+NV group that were sacrificed 3 days after I/R induction and **(D)** TUNEL staining was performed, and nuclei were counterstained with DAPI. **(E)** CMs were visualized by staining for cTnI. **(F)** The images were merged to identify TUNEL-positive CMs. **(G)** The proportion of CMs that were positive for TUNEL staining was calculated and presented as a percentage. The boxed regions in panels A-F were displayed at higher magnification in panels A1-F1, respectively. n=4 animals per group and values were presented as mean ± SD. Independent t-test: $^{**}P < 0.01$.

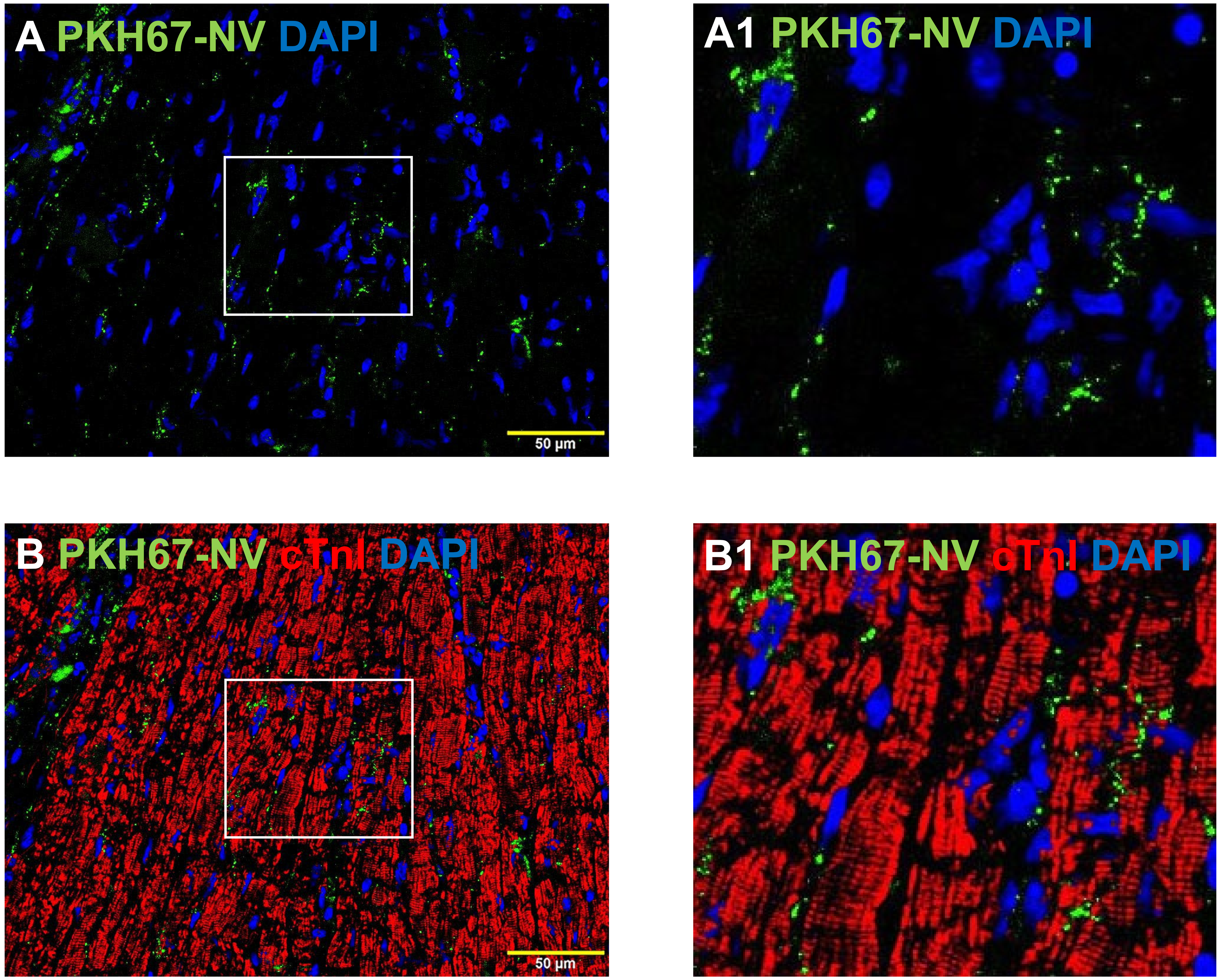

Supplemental Figure 19. [B2MKO]hiPSC-NVs were internalized by mouse CMs one week after I/R induction. To assess the internalization of [B2MKO]hiPSC-NVs by mouse CMs, [B2MKO]hiPSC-NVs were labelled with PKH67 (PKH67-NVs), a green fluorescent cell-binding dye, and injected into the hearts of mice after I/R induction. One week later, cryosections of mouse heart were stained with cardiac troponin I (cTnI) to identify mouse CMs. A representative image of PKH67-NVs **(A)** located in the mouse myocardium, with cTnI staining **(B)** used to identify mouse CMs. The selected areas were further magnified in panels A1 and B1, respectively.

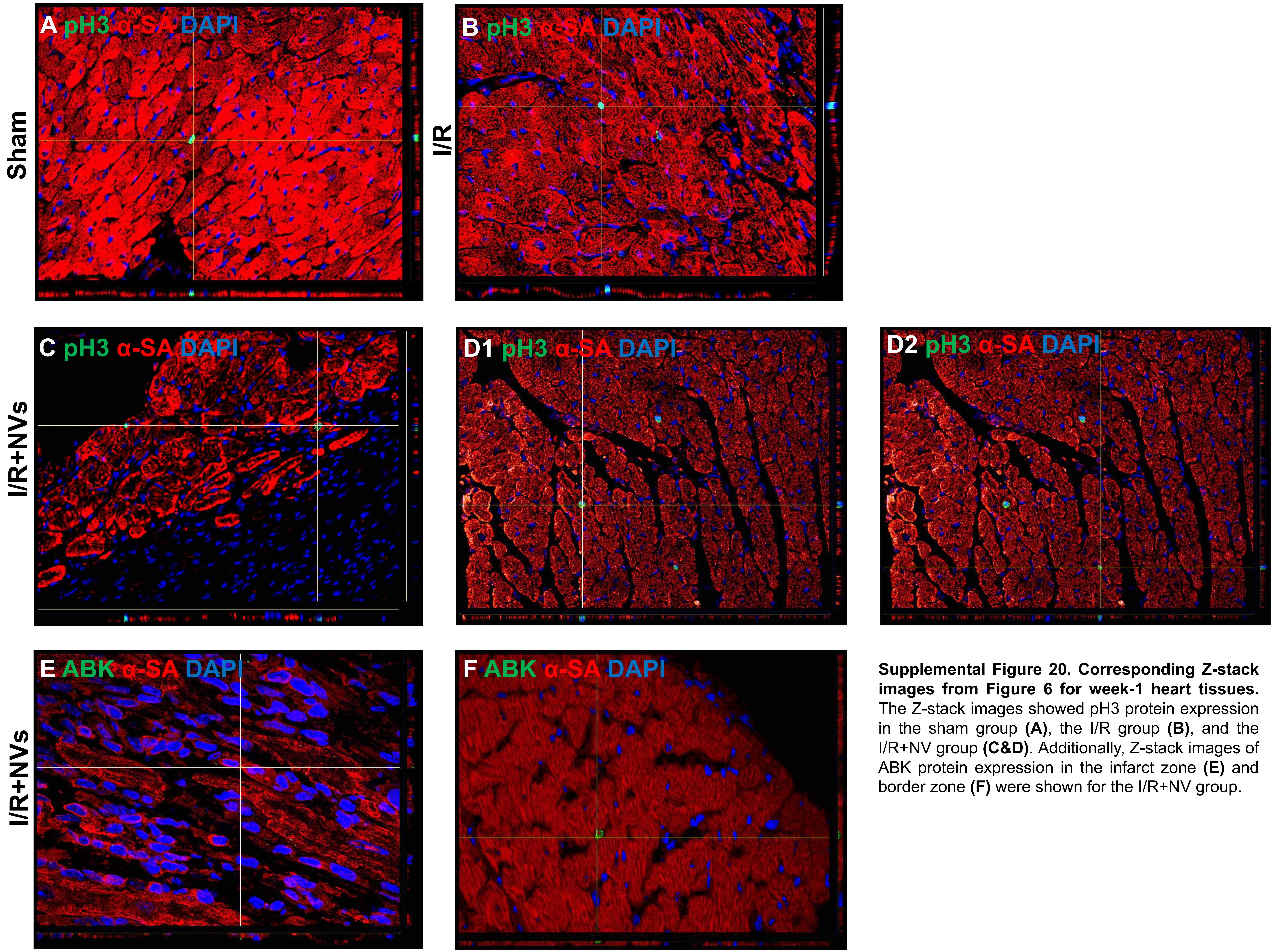

Supplemental Figure 20. Corresponding Z-stack images from Figure 6 for week-1 heart tissues. The Z-stack images showed pH3 protein expression in the sham group **(A)**, the I/R group **(B)**, and the I/R+NV group **(C&D)**. Additionally, Z-stack images of ABK protein expression in the infarct zone **(E)** and border zone **(F)** were shown for the I/R+NV group.

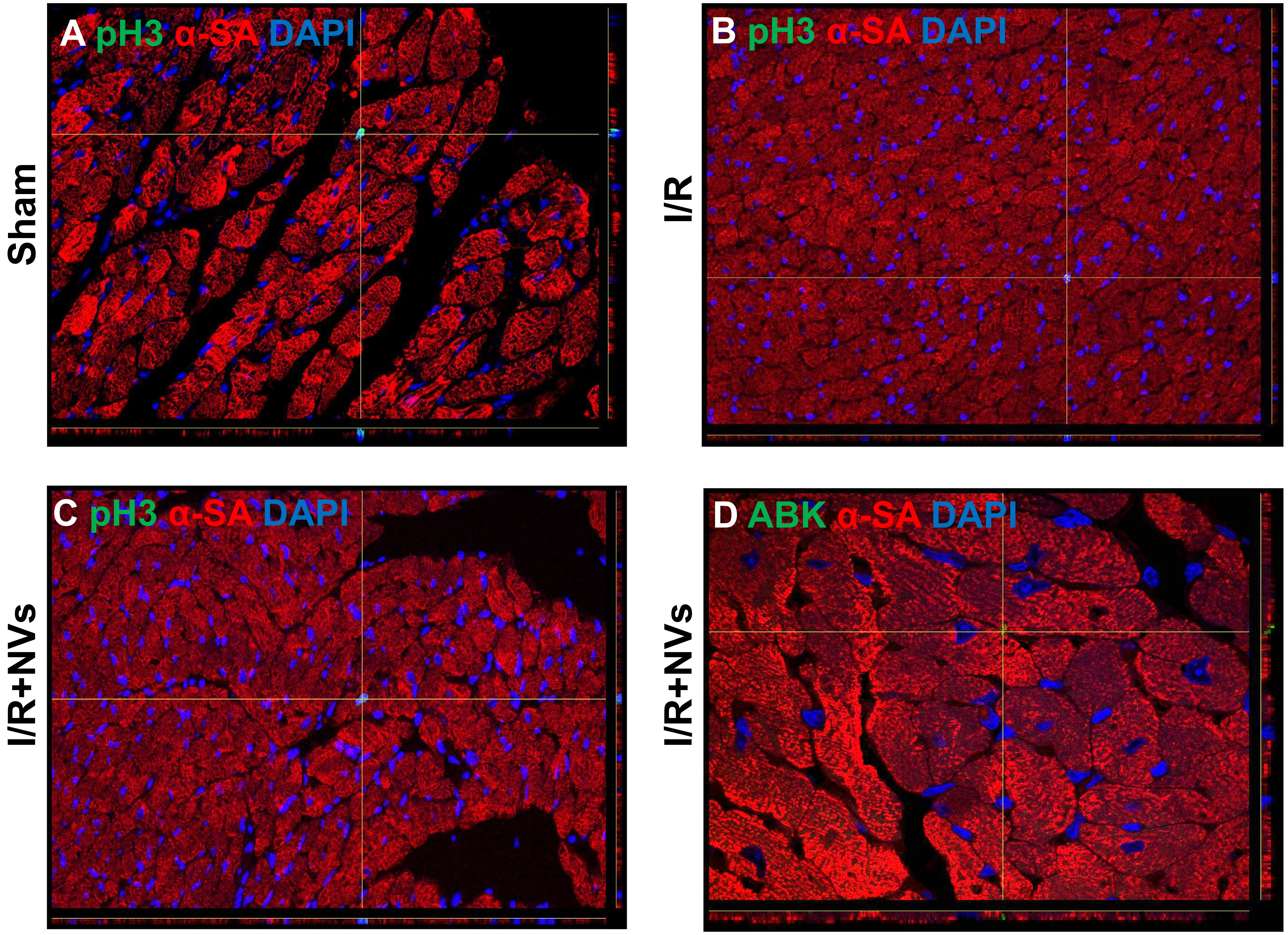

Supplemental Figure 21. Corresponding Z-stack images from Figure 6 for week-4 heart tissues. The Z-stack images showed pH3 protein expression in the sham group **(A)**, the I/R group **(B)**, and the I/R+NV group **(C)**. Additionally, Z-stack images of ABK protein expression in the border zone **(D)** were shown for the I/R+NV group.
