# Supplementary material for: Human induced pluripotent stem cell derived nanovesicles for cardiomyocyte protection and proliferation
Source: Bioact Mater. 2025 May 2;50:585–602. doi: 10.1016/j.bioactmat.2025.04.017 (PMC12124652; doi:10.1016/j.bioactmat.2025.04.017)
Supplement: Multimedia component 2 [file mmc2.pdf]

Table S1: Primers to check off-target sites for proximal guided RNA

Site	Sequencing primer	PCR forward primer	PCR reverse primer
1	TGTCTCTATCTGGACAGCCCAC	AACTCCTGGCCTCAAGTGATCC	AGTAGAGTCTGGGCAACTGCAG
2	TTCCCCAGTTACCCCATGACAC	AGCAGAGAAGGAAAGGTGGGTC	GGTTTCACCATGTTAGCCAGGC
3	AGGAGAAACAGAGGGGAAGACC	TTCTCTCCCAGACAAGCTCACC	TAGCCAGGCTGGTCTCAAACTC
4	ACATGGAAGGGATTTCAGGCTG	ACCCAGGAAATTGGACCCTCAG	AAGGTCAAGCAATGTGCCAAGG
5	TTCACTTCAGGACCTAAAGTGC	TGTCCTTGAGTGCCCAGTGTAG	AACATTGCAGCCACAGGTGATG

Table S2: Primers to check off-target sites for distal guided RNA

Site	Sequencing primer	PCR forward primer	PCR reverse primer
1	TGCCTACATCAAGGAAACATGCAC	AAGAGACCTGCCACAGCTACAG	TCCCCAAGTCTCCTTTCACACC
2	AGCCTAACACTTTTTATTCGGCC	AAGTGAACCCTTCCCTGTGGAG	TGGGTGGAGTGTAAGTGGTGTG
3	TGGGACTTCTGAGGTACACTTGC	ACACAGAGCTCTGATCCCTTGG	GAGTGTTGCTCAGTTGCATCCC
4	CCACCCATTCGTTCATTCAACAAG	AGCTTTTCACTGCAATGCTGTC	TGCAGCTCCATTCACTAGGACC
5	TGAGTCCTTGACTGTAGCTGCC	CACGGAATGGCACCAGTCAAAG	TTCCATCCTTCAGACCAGGCTG

Supplemental Table 3. Antibodies used for flow cytometry

Primary antibody	Volume	Category number	Company
Mouse anti-β2 microglobulin-FITC	5 µl	395706	Biolegend
Mouse anti-HLA-A/B/C-PE	5 µl	311406	Biolegend
Mouse anti-HLA-E-APC	5 µl	342606	Biolegend
Mouse anti-HLA-G-APC	5 µl	335909	Biolegend
Mouse anti-HLA-DR/DQ/DP-FITC	5 µl	361706	Biolegend
Mouse anti CD47-FITC	5 µl	323106	Biolegend
Mouse anti-CD38-APC	5 µl	303510	Biolegend
Mouse anti-CD69-PE	5 µl	310906	Biolegend
Rat anti-mouse CD3-PE	5 µl	100206	Biolegend
Rat anti-mouse CD4-FITC	5 µl	100406	Biolegend
Rat anti-mouse CD8a-FITC	5 µl	35-0081	Tonbo Biosciences
Hamster anti-mouse CD69-PE	5 µl	104510	Biolegend

Supplemental Table 4. Antibodies used in Western Blot analysis

Primary antibody	Dilution	Category NO.	Company	2nd antibody	Dilution	Company
Rabbit anti phosphorylated ERK1/2 (Thr202/Tyr204)	1:1000	4370S	Cell signaling	Goat anti-rabbit-IgG-HRP	1:4000	Cell signaling
Rabbit anti ERK1/2	1:1000	9102S	Cell signaling	Goat anti-rabbit-IgG-HRP	1:4000	Cell signaling
Rabbit anti phosphorylated P38MAPK (Thr180/Tyr182)	1:1000	4511S	Cell signaling	Goat anti-rabbit-IgG-HRP	1:4000	Cell signaling
Rabbit anti P38MAPK	1:1000	9212S	Cell signaling	Goat anti-rabbit-IgG-HRP	1:4000	Cell signaling
Rabbit antiphosphorylated AKT(S473)	1:1000	4060S	Cell signaling	Goat anti-rabbit-IgG-HRP	1:4000	Cell signaling
Rabbit anti AKT	1:1000	9272S	Cell signaling	Goat anti-rabbit-IgG-HRP	1:4000	Cell signaling
Rabbit anti GAPDH	1:5000	2118S	Cell signaling	Goat anti-rabbit-IgG-HRP	1:10000	Cell signaling
Rabbit anti-B2M	1:1000	ab218230	Abcam	Goat anti-rabbit-IgG-HRP	1:4000	Cell signaling
Rabbit anti-CIITA	1:400	ab7541	Abcam	Goat anti-rabbit-IgG-HRP	1:1000	Cell signaling
Rabbit anti-phosphorylated YAP (ser127)	1:500	13008S	Cell signaling	Goat anti-rabbit-IgG-HRP	1:2000	Cell signaling
Rabbit anti-YAP	1:500	14074S	Cell signaling	Goat anti-rabbit-IgG-HRP	1:2000	Cell signaling
Mouse anti-PRDX2	1:500	SC-515428	Santa Cruz	Goat anti-mouse IgG HRP	1:2000	Perkin Elmer
Mouse anti-PRDX6	1:100	SC-166454	Santa Cruz	Goat anti-mouse IgG HRP	1:500	Perkin Elmer
Mouse anti-GSR	1:500	SC-133245	Santa Cruz	Goat anti-mouse-IgG-HRP	1:2000	Perkin Elmer
Mouse anti-TPR	1:100	SC-101294	Santa Cruz	Goat anti-mouse IgG HRP	1:500	Perkin Elmer
Mouse anti-SKA2	1:500	SC-514495	Santa Cruz	Goat anti-mouse-IgG-HRP	1:2000	Perkin Elmer
Mouse anti-HP1α (CBX5)	1:100	SC-130446	Santa Cruz	Goat anti-mouse IgG HRP	1:500	Perkin Elmer
Mouse anti-INCENP	1:100	SC-376514	Santa Cruz	Goat anti-mouse IgG HRP	1:500	Perkin Elmer
Mouse anti-CKS1/2	1:100	SC-376663	Santa Cruz	Goat anti-mouse IgG HRP	1:500	Perkin Elmer
Rabbit anti-Ki67	1:100	ab16667	Abcam	Goat anti-rabbit IgG HRP	1:500	Perkin Elmer
Mouse anti-RIF1	1:100	SC-515573	Santa Cruz	Goat anti-mouse IgG HRP	1:500	Perkin Elmer
Mouse anti-RBBP4/7	1:500	SC-373873	Santa Cruz	Goat anti-mouse IgG HRP	1:1000	Perkin Elmer
